# Perturbation bounds for Monte Carlo within Metropolis via restricted approximations

**DOI:** 10.1016/j.spa.2019.06.015

**Published:** 2020-04

**Authors:** Felipe Medina-Aguayo, Daniel Rudolf, Nikolaus Schweizer

**Affiliations:** a Department of Mathematics and Statistics, University of Reading Whiteknights, PO Box 220, Reading RG6 6AX, United Kingdom; b Institute for Mathematical Stochastics, Universität Göttingen & Felix-Bernstein-Institute for Mathematical Statistics, Goldschmidtstraße 3-5, 37077 Göttingen, Germany; c Department of Econometrics and OR, Tilburg University, PO Box 90153, 5000 LE Tilburg, The Netherlands

**Keywords:** Markov chain Monte Carlo, Restricted approximation, Monte Carlo within Metropolis, Intractable likelihood

## Abstract

The Monte Carlo within Metropolis (MCwM) algorithm, interpreted as a perturbed Metropolis–Hastings (MH) algorithm, provides an approach for approximate sampling when the target distribution is intractable. Assuming the unperturbed Markov chain is geometrically ergodic, we show explicit estimates of the difference between the nth step distributions of the perturbed MCwM and the unperturbed MH chains. These bounds are based on novel perturbation results for Markov chains which are of interest beyond the MCwM setting. To apply the bounds, we need to control the difference between the transition probabilities of the two chains and to verify stability of the perturbed chain.

## 1 Introduction

The *Metropolis–Hastings* (MH) algorithm is a classical method for sampling approximately from a distribution of interest relying only on point-wise evaluations of an *unnormalized* density. However, when even this unnormalized density depends on unknown integrals and cannot easily be evaluated, then this approach is not feasible. A possible solution is to replace the required density evaluations in the MH acceptance ratio with suitable approximations. This idea is implemented in *Monte Carlo within Metropolis* (MCwM) algorithms which substitute the unnormalized density evaluations by Monte Carlo estimates for the intractable integrals.

Yet in general, replacing the exact MH acceptance ratio by an approximation leads to inexact algorithms in the sense that a stationary distribution of the transition kernel of the resulting Markov chain (if it exists) is not the distribution of interest. Moreover, convergence to a distribution is not at all clear. Nonetheless, these approximate, perturbed, or noisy methods, see e.g. [1], [10], [12], have recently gained increased attention due to their applicability in certain intractable sampling problems. In this work we attempt to answer the following questions about the MCwM algorithm:

• Can one quantify the quality of MCwM algorithms?
• When might the MCwM algorithm fail and what can one do in such situations?

Regarding the first question, by using bounds on the difference of the nth step distributions of a MH and a MCwM algorithm based Markov chain we give a positive answer. For the second question, we suggest a modification for stabilizing the MCwM approach by restricting the Markov chain to a suitably chosen set that contains the “essential part”, which we also call the “center” of the state space. We provide examples where this restricted version of MCwM converges towards the distribution of interest while the unrestricted version does not. Note also that in practical implementations of Markov chain Monte Carlo on a computer, simulated chains are effectively restricted to compact state spaces due to memory limitations. Our results on restricted approximations can also be read in this spirit.

**Perturbation theory.** Our overall approach is based on perturbation theory for Markov chains. Let ${\left(X_{n}\right)}_{n\in N_{0}}$ be a Markov chain with transition kernel P and ${\left({\overset{˜}{X}}_{n}\right)}_{n\in N_{0}}$ be a Markov chain with transition kernel $\overset{˜}{P}$ on a common Polish space (G,B(G)). We think of P and $\overset{˜}{P}$ as “close” to each other in a suitable sense and consider $\overset{˜}{P}$ as a perturbation of P. In order to quantify the difference of the distributions of $X_{n}$ and ${\overset{˜}{X}}_{n}$, denoted by $p_{n}$ and ${\overset{˜}{p}}_{n}$ respectively, we work with

(1) $${\left\|p_{n}-{\overset{˜}{p}}_{n}\right\|}_{\mathrm{tv}},$$

where ${\left\|\cdot \right\|}_{\mathrm{tv}}$ denotes the total variation distance. The Markov chain ${\left(X_{n}\right)}_{n\in N_{0}}$ can be interpreted as the unavailable, unperturbed, or ideal chain; while ${\left({\overset{˜}{X}}_{n}\right)}_{n\in N_{0}}$ is a perturbation that is available for simulation. We focus on the case where the ideal Markov chain is *geometrically ergodic*, more precisely V-*uniformly ergodic*, implying that its transition kernel P satisfies a *Lyapunov condition* of the form

PV(x)≤δV(x)+L,x∈G,

for some function V:G→[1,∞) and numbers δ∈[0,1),L∈[1,∞).

To obtain estimates of (1) we need two assumptions which can be informally explained as follows:

1. *Closeness of* $\overset{˜}{P}$ *and* P*:* The difference of $\overset{˜}{P}$ and P is measured by controlling either a weighted total variation distance or a weighted V-norm of $P\left(x,\cdot \right)-\overset{˜}{P}\left(x,\cdot \right)$ uniformly. Here, uniformity either refers to the entire state space or, at least, to the “essential” part of it.
2. *Stability of* $\overset{˜}{P}$*:* A stability condition on $\overset{˜}{P}$ is satisfied either in the form of a Lyapunov condition or by restriction to the center of the state space determined by V.

Under these assumptions, explicit bounds on (1) are provided in Section 3. More precisely, in Proposition 6 and Theorem 7 stability is guaranteed through a Lyapunov condition for $\overset{˜}{P}$, whereas in Theorem 9 a *restricted approximation* $\overset{˜}{P}$ is considered.

**Monte Carlo within Metropolis.** In Section 4 we apply our perturbation bounds in the context of approximate sampling via MCwM. In the following we briefly introduce the setting. The goal is to (approximately) sample from a target distribution π on G, which is determined by an unnormalized density function $\pi_{u}:G\to \left[0,\infty \right)$ w.r.t a reference measure μ, that is,

$$\pi \left(A\right)=\frac{\int_{A}\pi_{u}\left(x\right)\;d\mu \left(x\right)}{\int_{G}\pi_{u}\left(x\right)\;d\mu \left(x\right)},\;A\in B\left(G\right).$$

Classically the method of choice is to construct a Markov chain ${\left(X_{n}\right)}_{n\in N_{0}}$ based on the MH algorithm for approximate sampling of π. This algorithm crucially relies on knowing (at least) the ratio $\pi_{u}\left(y\right)∕\pi_{u}\left(x\right)$ for arbitrary $\left(x,y\right)\in G^{2}$, e.g., because $\pi_{u}\left(x\right)$ and $\pi_{u}\left(y\right)$ can readily be computed. However, in some scenarios, only approximations of $\pi_{u}\left(x\right)$ and $\pi_{u}\left(y\right)$ are available. Replacing the true unnormalized density $\pi_{u}$ in the MH algorithm by an approximation yields a perturbed, “inexact” Markov chain ${\left({\overset{˜}{X}}_{n}\right)}_{n\in N_{0}}$. If the approximation is based on a Monte Carlo method, the perturbed chain is called MCwM chain.

Two particular settings where approximations of $\pi_{u}$ may rely on Monte Carlo estimates are *doubly-intractable distributions* and *latent variables*. Examples of the former occur in Markov or Gibbs random fields, where the function values $\pi_{u}\left(x\right)$ of the unnormalized density itself are only known up to a factor Z(x). This means that

(2) $$\pi_{u}\left(x\right)=\rho \left(x\right)∕Z\left(x\right),\;x\in G,$$

where only values of ρ(x) can easily be computed while the computational problem lies in evaluating

**Figure:**
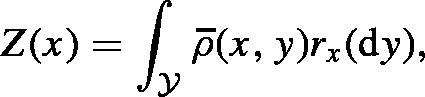

where Y denotes an auxiliary variable space,  and $r_{x}$ is a probability distribution on Y. We investigate a MCwM algorithm, which in every transition uses an iid sequence of random variables ${\left(Y_{i}^{\left(x\right)}\right)}_{1\leq i\leq N}$, with $Y_{1}^{\left(x\right)}∼r_{x}$, to approximate Z(x) by  (and Z(y) by Z^N(y), respectively). The second setting we study arises from *latent variables*. Here, $\pi_{u}\left(x\right)$ cannot be evaluated since it takes the form (3)
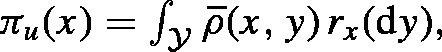
where $r_{x}$ is a probability distribution on a measurable space Y of latent variables y, and  is a non-negative density function. In general, no explicit computable expression of the above integral is at hand and the MCwM idea is to substitute $\pi_{u}\left(x\right)$ in the MH algorithm by a Monte Carlo estimate based on iid sequences of random variables ${\left(Y_{i}^{\left(x\right)}\right)}_{1\leq i\leq N}$ and ${\left(Y_{i}^{\left(y\right)}\right)}_{1\leq i\leq N}$ with $Y_{1}^{\left(x\right)}∼r_{x}$, $Y_{1}^{\left(y\right)}∼r_{y}$. The resulting MCwM algorithm has been studied before in [3], [14]. Let us note here that this MCwM approach should not be confused with the pseudo-marginal method, see [3]. The pseudo-marginal method constructs a Markov chain on the extended space G×Y that targets a distribution with π as its marginal on G.

**Perturbation bounds for MCwM.** In both intractability settings, the corresponding MCwM Markov chains depend on the parameter N∈N which denotes the number of samples used within the Monte Carlo estimates. As a consequence, any bound on (1) is N-dependent, which allows us to control the dissimilarity to the ideal MH based Markov chain. In Corollary 16 and the application of Corollary 17 to the examples considered in Section 4 we provide informative rates of convergence as N→∞. Note that with those estimates we relax the requirement of uniform bounds on the approximation error introduced by the estimator for $\pi_{u}$, which is essentially imposed in [1], [14]. In contrast to this requirement, we use (if available) the Lyapunov function as a counterweight for a second as well as inverse second moment and can therefore handle situations where uniform bounds on the approximation error are not available. If we do not have access to a Lyapunov function for the MCwM transition kernel we suggest to restrict it to a subset of the state space, i.e., use restricted approximations. This subset is determined by V and usually corresponds to a ball with some radius R(N) that increases as the approximation quality improves, that is, R(N)→∞ as N→∞.

Our analysis of the MCwM algorithm is guided by some facts we observe in simple illustrations, in particular, we consider a log-normal example discussed in Section 4.1. In this example, we encounter a situation where the mean squared error of the Monte Carlo approximation grows exponentially in the tail of the target distribution. We observe *empirically* that (unrestricted) MCwM works well whenever the growth behavior is dominated by the decay of the (Gaussian) target density in the tail. The application of Corollary 17 to the log-normal example shows that the restricted approximation converges towards the true target density in the number of samples N at least like ${\left(\log N\right)}^{-1}$ independent of *any* growth of the error. However, the convergence is better, at least like $\frac{\log N}{N}$, if the growth is dominated by the decay of the target density.

## 2 Preliminaries

Let G be a Polish space, where B(G) denotes its Borel σ-algebra. Assume that P is a transition kernel with stationary distribution π on G. For a signed measure q on G and a measurable function f:G→R we define

$$qP\left(A\right)≔\int_{G}P\left(x,A\right)\;dq\left(x\right),\;Pf\left(x\right)≔\int_{G}f\left(y\right)\;P\left(x,dy\right),\;x\in G,A\in B\left(G\right).$$

For a distribution μ on G we use the notation $\mu \left(f\right)≔\int_{G}f\left(x\right)\;d\mu \left(x\right).$ For a measurable function V:G→[1,∞) and two probability measures μ,ν on G define

$${\left\|\mu -\nu \right\|}_{V}≔\underset{\left|f\right|\leq V}{\sup}\left|\mu \left(f\right)-\nu \left(f\right)\right|.$$

For the constant function V=1 this is the total variation distance, i.e., 

$${\left\|\mu -\nu \right\|}_{\mathrm{tv}}≔\underset{\left|f\right|\leq 1}{\sup}\left|\mu \left(f\right)-\nu \left(f\right)\right|.$$

The next, well-known theorem defines geometric ergodicity and states a useful equivalent condition. The proof follows by [23, Proposition 2.1] and [17, Theorem 16.0.1].

Theorem 1 *For a* ϕ*-irreducible and aperiodic transition kernel* P *with stationary distribution* π *defined on* G *the following statements are equivalent:*

• *The transition kernel* P *is* geometrically ergodic*, that is, there exists a number* $\overset{¯}{\alpha }\in \left[0,1\right)$ *and a measurable function* C:G→[1,∞) *such that for* π*-a.e.* x∈G *we have*
  (4) $${\left\|P^{n}\left(x,\cdot \right)-\pi \right\|}_{\mathrm{tv}}\leq C\left(x\right){\overset{¯}{\alpha }}^{n},\;n\in N.$$
• *There is a* π*-a.e. finite measurable function* V:G→[1,∞] *with finite moments with respect to* π *and there are constants* α∈[0,1) *and* C∈[1,∞) *such that*
  (5) $${\left\|P^{n}\left(x,\cdot \right)-\pi \right\|}_{V}\leq CV\left(x\right)\alpha^{n},\;x\in G,\;n\in N.$$

*In particular, the function* V *can be chosen such that a* Lyapunov condition *of the form*

(6) PV(x)≤δV(x)+L,x∈G,

*for some* δ∈[0,1) *and* L∈(0,∞)*, is satisfied.*

Remark 2 We call a transition kernel V-*uniformly ergodic* if it satisfies (5) and note that this condition can be rewritten as

(7) $$\underset{x\in G}{\sup}\frac{{\left\|P^{n}\left(x,\cdot \right)-\pi \right\|}_{V}}{V\left(x\right)}\leq C\alpha^{n}.$$

## 3 Quantitative perturbation bounds

Assume that ${\left(X_{n}\right)}_{n\in N_{0}}$ is a Markov chain with transition kernel P and initial distribution $p_{0}$ on G. We define $p_{n}≔p_{0}P^{n}$, i.e., $p_{n}$ is the distribution of $X_{n}$. The distribution $p_{n}$ is approximated by using another Markov chain ${\left({\overset{˜}{X}}_{n}\right)}_{n\in N_{0}}$ with transition kernel $\overset{˜}{P}$ and initial distribution ${\overset{˜}{p}}_{0}$. We define ${\overset{˜}{p}}_{n}≔{\overset{˜}{p}}_{0}{\overset{˜}{P}}^{n}$, i.e., ${\overset{˜}{p}}_{n}$ is the distribution of ${\overset{˜}{X}}_{n}$. The idea throughout the paper is to interpret ${\left(X_{n}\right)}_{n\in N_{0}}$ as some ideal, unperturbed chain and ${\left({\overset{˜}{X}}_{n}\right)}_{n\in N_{0}}$ as an approximating, perturbed Markov chain.

In the spirit of the doubly-intractable distribution and latent variable case considered in Section 4 we think of the unperturbed Markov chain as “nice”, where convergence properties are readily available. Unfortunately since we cannot simulate the “nice” chain we try to approximate it with a perturbed Markov chain, which is, because of the perturbation, difficult to analyze directly. With this in mind, we make the following standing assumption on the unperturbed Markov chain.

Assumption 3 Let V:G→[1,∞) be a measurable function and assume that P is V-uniformly ergodic, that is, (5) holds for some constants C∈[1,∞) and α∈[0,1).

We start with an auxiliary estimate of ${\left\|p_{n}-{\overset{˜}{p}}_{n}\right\|}_{\mathrm{tv}}$ which is interesting on its own and is proved in Appendix A.1.

Lemma 4 *Let* Assumption 3 *be satisfied and for a measurable function*W:G→[1,∞) *define*

$$\varepsilon_{\mathrm{tv},W}≔\underset{x\in G}{\sup}\frac{{\left\|P\left(x,\cdot \right)-\overset{˜}{P}\left(x,\cdot \right)\right\|}_{\mathrm{tv}}}{W\left(x\right)},$$

$$\varepsilon_{V,W}≔\underset{x\in G}{\sup}\frac{{\left\|P\left(x,\cdot \right)-\overset{˜}{P}\left(x,\cdot \right)\right\|}_{V}}{W\left(x\right)}.$$

*Then, for any* r∈(0,1]*,*

(8) $${\left\|p_{n}-{\overset{˜}{p}}_{n}\right\|}_{\mathrm{tv}}\leq C\alpha^{n}{\left\|p_{0}-{\overset{˜}{p}}_{0}\right\|}_{V}+\varepsilon_{\mathrm{tv},W}^{1-r}\;\varepsilon_{V,W}^{r}\;C^{r}\overset{n-1}{\underset{i=0}{\sum }}{\overset{˜}{p}}_{i}\left(W\right)\alpha^{\left(n-i-1\right)r}.$$

Remark 5 The quantities $\varepsilon_{\mathrm{tv},W}$ and $\varepsilon_{V,W}$ measure the difference between P and $\overset{˜}{P}$. Note that we can interpret them as operator norms

$$\varepsilon_{\mathrm{tv},W}={\left\|P-\overset{˜}{P}\right\|}_{B^{\left(1\right)}\to B^{\left(W\right)}}\;\text{and}\;\varepsilon_{V,W}={\left\|P-\overset{˜}{P}\right\|}_{B^{\left(V\right)}\to B^{\left(W\right)}},$$

where

(9) $$B^{\left(W\right)}=\left\{f:G\to R|{\left\|f\right\|}_{\infty ,W}≔\underset{x\in G}{\sup}\frac{\left|f\left(x\right)\right|}{W\left(x\right)}<\infty \right\}.$$

It is also easily seen that $\varepsilon_{\mathrm{tv},W}\leq \min\left\{2,\varepsilon_{V,W}\right\}$ which implies that a small number $\varepsilon_{V,W}$ leads also to a small number $\varepsilon_{\mathrm{tv},W}$. In (8) an additional parameter r appears which can be used to tune the estimate. Namely, if one is not able to bound $\varepsilon_{V,W}$ sufficiently well but has a good estimate of $\varepsilon_{\mathrm{tv},W}$ one can optimize over r. On the other hand, if there is a satisfying estimate of $\varepsilon_{V,W}$ one can just set r=1.

In the previous lemma we proved an upper bound of ${\left\|p_{n}-{\overset{˜}{p}}_{n}\right\|}_{\mathrm{tv}}$ which still contains an unknown quantity given by

$$\overset{n-1}{\underset{i=0}{\sum }}{\overset{˜}{p}}_{i}\left(W\right)\alpha^{\left(n-i-1\right)r}$$

which measures, in a sense, stability of the perturbed chain through a weighted sum of expectations of the Lyapunov function W under ${\overset{˜}{p}}_{i}$. To control this term, we impose additional assumptions on the perturbed chain. In the following, we consider two assumptions of this type, a Lyapunov condition and a bounded support assumption.

### 3.1 Lyapunov condition

We start with a simple version of our main estimate which illustrates already some key aspects of the approach via the Lyapunov condition. Here the intuition is as follows: By Theorem 1 we know that the function V of Assumption 3 can be chosen such that a Lyapunov condition for P is satisfied. Since we think of $\overset{˜}{P}$ as being close to P, it might be possible to show also a Lyapunov condition with V of $\overset{˜}{P}$. If this is the case, the following proposition is applicable.

Proposition 6 *Let* Assumption 3 *be satisfied. Additionally, let* $\overset{˜}{\delta }\in \left[0,1\right)$ *and* $\overset{˜}{L}\in \left(0,\infty \right)$ *be such that*

(10) $$\overset{˜}{P}V\left(x\right)\leq \overset{˜}{\delta }\;V\left(x\right)+\overset{˜}{L},\;x\in G.$$

*Assume that* $p_{0}={\overset{˜}{p}}_{0}$ *and define* $\kappa ≔\max\left\{{\overset{˜}{p}}_{0}\left(V\right),\frac{\overset{˜}{L}}{1-\overset{˜}{\delta }}\right\},$ *as well as (for simplicity)*

$$\varepsilon_{\mathrm{tv}}≔\varepsilon_{\mathrm{tv},V},\;\varepsilon_{V}≔\varepsilon_{V,V}.$$

*Then, for any* r∈(0,1]*,*

(11) $${\left\|p_{n}-{\overset{˜}{p}}_{n}\right\|}_{\text{tv}}\leq \varepsilon_{\text{tv}}^{1-r}\varepsilon_{V}^{r}\;\frac{C^{r}\kappa }{\left(1-\alpha \right)r}.$$

Proof We use Lemma 4 with W=V. By (10), it follows that

(12) $${\overset{˜}{p}}_{i}\left(V\right)=\int_{G}{\overset{˜}{P}}^{i}V\left(x\right)\;{\overset{˜}{p}}_{0}\left(dx\right)\leq {\overset{˜}{\delta }}^{i}{\overset{˜}{p}}_{0}\left(V\right)+\left(1-{\overset{˜}{\delta }}^{i}\right)\frac{\overset{˜}{L}}{1-\overset{˜}{\delta }}\leq \kappa .$$

The final estimate is obtained by a geometric series and $1-\alpha^{r}\geq r\left(1-\alpha \right)$. □

Now we state a more general theorem. In particular, in this estimate the dependence on the initial distribution can be weakened. In the perturbation bound of the previous estimate, the initial distribution is only forgotten if ${\overset{˜}{p}}_{0}\left(V\right)<\overset{˜}{L}∕\left(1-\overset{˜}{\delta }\right)$. Yet, intuitively, for long-term stability results ${\overset{˜}{p}}_{0}\left(V\right)$ should not matter at all. This intuition is confirmed by the theorem.

Theorem 7 *Let* Assumption 3 *be satisfied. Assume also that*W:G→[1,∞) *is a measurable function which satisfies with* $\overset{˜}{\delta }\in \left[0,1\right)$ *and* $\overset{˜}{L}\in \left(0,\infty \right)$ *the Lyapunov condition*

(13) $$\overset{˜}{P}W\left(x\right)\leq \overset{˜}{\delta }W\left(x\right)+\overset{˜}{L},\;x\in G.$$

*Define* $\varepsilon_{\mathrm{tv},W}$*,*$\varepsilon_{V,W}$ *as in* Lemma 4 *and* $\gamma ≔\frac{\overset{˜}{L}}{1-\overset{˜}{\delta }}$*. Then, for any* r∈(0,1] *with*

$$\beta_{n,r}\left(\overset{˜}{\delta },\alpha \right)≔\left\{\begin{array}{cc} n\alpha^{\left(n-1\right)r},\; & \alpha^{r}=\overset{˜}{\delta },\\ \frac{\left|\alpha^{rn}-{\overset{˜}{\delta }}^{n}\right|}{\left|\alpha^{r}-\delta \right|},\; & \alpha^{r}\neq \overset{˜}{\delta }, \end{array}\right)$$

*we have*

(14) $${\left\|{\overset{˜}{p}}_{n}-p_{n}\right\|}_{\mathrm{tv}}\leq C\alpha^{n}{\left\|{\overset{˜}{p}}_{0}-p_{0}\right\|}_{V}+\varepsilon_{\mathrm{tv},W}^{1-r}\;\varepsilon_{V,W}^{r}\;C^{r}\left[{\overset{˜}{p}}_{0}\left(W\right)\;\beta_{n,r}\left(\overset{˜}{\delta },\alpha \right)+\frac{\gamma }{\left(1-\alpha \right)r}\right].$$

Proof Here we use Lemma 4 with possibly different W and V. By (13) we have ${\overset{˜}{p}}_{i}\left(W\right)\leq {\overset{˜}{\delta }}^{i}{\overset{˜}{p}}_{0}\left(W\right)+\gamma$ and by

$$\overset{n-1}{\underset{i=0}{\sum }}{\overset{˜}{\delta }}^{i}\alpha^{\left(n-i-1\right)r}=\beta_{n,r}\left(\overset{˜}{\delta },\alpha \right)$$

we obtain the assertion by a geometric series and $1-\alpha^{r}\geq r\left(1-\alpha \right)$. □

Remark 8 We consider an illustrating example where Theorem 7 leads to a considerably sharper bound than Proposition 6. This improvement is due to the combination of two novel properties of the bound of Theorem 7:

1. In the Lyapunov condition (13) the function W can be chosen differently from V.
2. Note that $\beta_{n,r}\left(\overset{˜}{\delta },\alpha \right)$ is bounded from above by $n\cdot \max{\left\{\overset{˜}{\delta },\alpha^{r}\right\}}^{n-1}$. Thus $\beta_{n,r}\left(\overset{˜}{\delta },\alpha \right)$ converges almost exponentially fast to zero in n. This implies that for n sufficiently large the dependence of ${\overset{˜}{p}}_{0}\left(W\right)$ vanishes. Nevertheless, the leading factor n can capture situations in which the perturbation error is increasing in n for small n.

**Illustrating example.** Let G={0,1} and assume $p_{0}={\overset{˜}{p}}_{0}=\left(0,1\right)$. Here state “1” can be interpreted as “transitional” while state “0” as “essential” part of the state space. Define

$$P=\left(\begin{array}{cc} 1 & 0\\ 1 & 0 \end{array}\right)\;\text{and}\;\overset{˜}{P}=\left(\begin{array}{cc} 1 & 0\\ \frac{1}{2} & \frac{1}{2} \end{array}\right).$$

Thus, the unperturbed Markov chain ${\left(X_{n}\right)}_{n\in N_{0}}$ moves from “1” to “0” right away, while the perturbed one ${\left({\overset{˜}{X}}_{n}\right)}_{n\in N_{0}}$ takes longer. Both transition matrices have the same stationary distribution π=(1,0). Obviously, ${\left\|p_{0}-{\overset{˜}{p}}_{0}\right\|}_{\mathrm{tv}}=0$ and for n∈N it holds that

$${\left\|p_{n}-{\overset{˜}{p}}_{n}\right\|}_{\mathrm{tv}}=2P\left(X_{n}\neq {\overset{˜}{X}}_{n}\right)=\frac{1}{2^{n-1}}.$$

The unperturbed Markov chain is uniformly ergodic, such that we can choose V=1 and (5) is satisfied with C=1 and α=0. In particular, in this setting $\varepsilon_{\mathrm{tv}}$ and $\varepsilon_{V}$ from Proposition 6 coincide, we have $\varepsilon_{\mathrm{tv}}=1$. Thus, the estimate of Proposition 6 gives

$${\left\|p_{n}-{\overset{˜}{p}}_{n}\right\|}_{\mathrm{tv}}\leq \varepsilon_{\mathrm{tv}}=1.$$

This bound is optimal in the sense that it is best possible for n=1. But for increasing n it is getting worse. Notice also that a different choice of V cannot really remedy this situation: The chains differ most strongly at n=1 and the bound of Proposition 6 is constant over time. Now choose the function $W\left(x\right)=1+v\cdot 1_{\left\{x=1\right\}}$ for some v≥0. The transition matrix $\overset{˜}{P}$ satisfies the Lyapunov condition

$$\overset{˜}{P}W\left(x\right)\leq \frac{1}{2}\;W\left(x\right)+\frac{1}{2},$$

i.e., $\overset{˜}{\delta }=\overset{˜}{L}=\frac{1}{2}$. Moreover, we have ${\overset{˜}{p}}_{0}\left(W\right)=1+v$ and $\varepsilon_{V,W}=\varepsilon_{\mathrm{tv},W}=1∕\left(1+v\right)$. Thus, in the bound from Theorem 7 we can set r=1 and γ=1 such that

$${\left\|p_{n}-{\overset{˜}{p}}_{n}\right\|}_{\mathrm{tv}}\leq \frac{1}{v+1}+\frac{1}{2^{n-1}}.$$

Since v can be chosen arbitrarily large, it follows that

$${\left\|p_{n}-{\overset{˜}{p}}_{n}\right\|}_{\mathrm{tv}}\leq \frac{1}{2^{n-1}},$$

which is best possible for all n∈N.

The previous example can be seen as a toy model of a situation where the transition probabilities of a perturbed and unperturbed Markov chain are very similar in the “essential” part of the state space, but differ considerably in the “tail”, seen as the “transitional” part. When the chains start both at the same point in the “tail”, considerable differences between distributions can build up along the initial transient and then vanish again. Earlier perturbation bounds as for example in [18], [22], [26] take only an initial error and a remaining error into account. Thus, those are worse for situations where this transient error captured by $\beta_{n,r}$ dominates. A very similar term also appears in the very recent error bounds due to [10]. In any case, the example also illustrates that a function W different from V is advantageous.

### 3.2 Restricted approximation

In the previous section, we have seen that a Lyapunov condition of the perturbation helps to control the long-term stability of approximating a V-uniformly ergodic Markov chain. In this section we assume that the perturbed chain is restricted to a “large” subset of the state space. In this setting a sufficiently good approximation of the unperturbed Markov chain on this subset leads to a perturbation estimate.

For the unperturbed Markov chain we assume that transition kernel P is V-uniformly ergodic. Then, for R≥1 define the “large subset” of the state space as

$$B_{R}=\left\{x\in G|V\left(x\right)\leq R\right\}.$$

If V is chosen as a monotonic transformation of a norm on G, $B_{R}$ is simply a ball around 0. The *restriction of* P to the set $B_{R}$, given as $P_{R}$, is defined as

$$P_{R}\left(x,A\right)=P\left(x,A\cap B_{R}\right)+1_{A}\left(x\right)P\left(x,B_{R}^{c}\right),\;A\in B\left(G\right),\;x\in G.$$

In other words, whenever P would make a transition from $x\in B_{R}$ to $G\setminus B_{R}$, $P_{R}$ remains in x. Otherwise, $P_{R}$ is the same as P. We obtain the following perturbation bound for approximations whose stability is guaranteed through a restriction to the set $B_{R}$.

Theorem 9 *Under the* V*-uniform ergodicity of* Assumption 3 *let* δ∈[0,1) *and* L∈[1,∞) *be chosen in such a way that*

PV(x)≤δV(x)+L,x∈G.

*For the perturbed transition kernel* $\overset{˜}{P}$ *assume that it is restricted to* $B_{R}$*, i.e.,* $\overset{˜}{P}\left(x,B_{R}\right)=1$ *for all*x∈G*, and that* R⋅Δ(R)≤(1−δ)∕2 *with*

$$\Delta \left(R\right)≔\underset{x\in B_{R}}{\sup}\frac{{\left\|P_{R}\left(x,\cdot \right)-\overset{˜}{P}\left(x,\cdot \right)\right\|}_{\mathrm{tv}}}{V\left(x\right)}.$$

*Then, with* $p_{0}={\overset{˜}{p}}_{0}$ *and*

$$\kappa ≔\max\left\{{\overset{˜}{p}}_{0}\left(V\right),\frac{L}{1-\delta }\right\}$$

*we have for* R≥exp(1) *that*

(15) $${\left\|p_{n}-{\overset{˜}{p}}_{n}\right\|}_{\mathrm{tv}}\leq \frac{33C\left(L+1\right)\kappa }{1-\alpha }\cdot \frac{\log R}{R}.$$

The proof of the result is stated in Appendix A.1. Notice that while the perturbed chain is restricted to the set $B_{R}$, we do not place a similar restriction on the unperturbed chain. The estimate (15) compares the restricted, perturbed chain to the unrestricted, unperturbed one.

Remark 10 In the special case where $\overset{˜}{P}\left(x,\cdot \right)=P_{R}\left(x,\cdot \right)$ for $x\in B_{R}$ we have Δ(R)=0. For example

$$\overset{˜}{P}\left(x,A\right)=1_{B_{R}}\left(x\right)P_{R}\left(x,A\right)+1_{B_{R}^{c}}\left(x\right)\delta_{x_{0}}\left(A\right),\;A\in B\left(G\right),$$

with $x_{0}\in B_{R}$ satisfies this condition. The resulting perturbed Markov chain is simply a restriction of the unperturbed Markov chain to $B_{R}$ and Theorem 9 provides a quantitative bound on the difference of the distributions.

### 3.3 Relationship to earlier perturbation bounds

In contrast to the V-uniform ergodicity assumption we impose on the ideal Markov chain, the results in [1], [12], [18] only cover perturbations of uniformly ergodic Markov chains. Nonetheless, perturbation theoretical questions for geometrically ergodic Markov chains have been studied before, see e.g. [5], [7], [14], [20], [24], [26], [28] and the references therein. A crucial aspect where those papers differ from each other is how one measures the closeness of the transitions of the unperturbed and perturbed Markov chains to have applicable estimates, see the discussion about this in [7], [26], [28]. Our Proposition 6 and Theorem 7 refine and extend the results of [26, Theorem 3.2]. In particular, in Theorem 7 we take a restriction to the center of the state space into account. Let us also mention here that [22], [26] contain related results under Wasserstein ergodicity assumptions. More recently, [11] studies approximate chains using notions of maximal couplings, [20] extends the uniformly ergodic setting from [12] to using $L_{2}$ norms instead of total variation, and [10] explores bounds on the approximation error of time averages.

The usefulness of restricted approximations in the study of Markov chains has been observed before. For example in [27], in an infinite-dimensional setting, spectral gap properties of a Markov operator based on a restricted approximation are investigated. Also recently in [30] it is proposed to consider a subset of the state space termed “large set” in which a certain Lyapunov condition holds. This is in contrast to a Lyapunov function defined on the entire space, which might deteriorate as the dimension of the state space or the number of observations increases. This new Lyapunov condition from [30] is particularly useful for obtaining explicit bounds on the number of iterations to get close to the stationary distribution in high-dimensional settings.

## 4 Monte Carlo within Metropolis

In Bayesian statistics it is of interest to sample with respect to a distribution π on (G,B(G)). We assume that π admits a possibly *unnormalized density* $\pi_{u}:G\to \left[0,\infty \right)$ with respect to a reference measure μ, for example the counting, Lebesgue or some Gaussian measure. The Metropolis–Hastings (MH) algorithm is often the method of choice to draw approximate samples according to π:

Algorithm 1 For a *proposal transition kernel* Q a transition from x to y of the MH algorithm works as follows.

1. Draw U∼Unif[0,1] and a proposal Z∼Q(x,⋅) independently, call the result u and z, respectively.
2. Compute the *acceptance ratio*
  (16) $$r\left(x,z\right)≔\frac{\pi \left(dz\right)Q\left(z,dx\right)}{\pi \left(dx\right)Q\left(x,dz\right)}=\frac{\pi_{u}\left(z\right)}{\pi_{u}\left(x\right)}\frac{\mu \left(dz\right)Q\left(z,dx\right)}{\mu \left(dx\right)Q\left(x,dz\right)},$$
  which is the density of the measure π(dz)Q(z,dx) w.r.t. π(dx)Q(x,dz), see [29].
3. If u<r(x,z), then accept the proposal, and return y≔z, otherwise reject the proposal and return y≔x.

The transition kernel of the MH algorithm with proposal Q, stationary distribution π and acceptance probability

$$a\left(x,z\right)≔\min\left\{1,r\left(x,z\right)\right\}$$

is given by

(17) $$M_{a}\left(x,dz\right)≔a\left(x,z\right)Q\left(x,dz\right)+\delta_{x}\left(dz\right)\left(1-\int_{G}a\left(x,y\right)Q\left(x,dy\right)\right).$$

For the MH algorithm in the computation of r(x,z) one uses $\pi_{u}\left(z\right)∕\pi_{u}\left(x\right)$, which might be known from having access to function evaluations of the unnormalized density $\pi_{u}$. However, when it is expensive or even impossible to compute function values of $\pi_{u}$, then it may not be feasible to sample from π using the MH algorithm. Here are two typical examples of such scenarios:

• **Doubly-intractable distribution:** For models such as *Markov or Gibbs random fields*, the unnormalized density $\pi_{u}\left(x\right)$ itself is typically only known up to a factor Z(x), that is,
  (18) $$\pi_{u}\left(x\right)=\rho \left(x\right)∕Z\left(x\right),\;x\in G$$
  where functions values of ρ can be computed, but function values of Z cannot. For instance, Z might be given in the form

**Figure:**
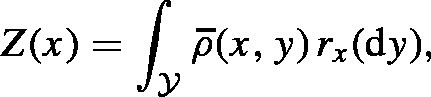

where Y denotes an auxiliary variable space,  and $r_{x}$ is a probability distribution on Y.
• **Latent variables:** Here $\pi_{u}\left(x\right)$ cannot be evaluated, since it takes the form (19)
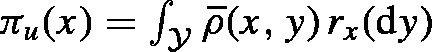
with a probability distribution $r_{x}$ on a measurable space Y of *latent variables* y and a non-negative function  .

In the next sections, we study in both of these settings the perturbation error of an approximating MH algorithm. A fair assumption in both scenarios, which holds for a large family of target distributions using random-walk type proposals, see, e.g., [9], [16], [25], is that the infeasible, unperturbed MH algorithm is V-uniformly ergodic:

Assumption 11 For some function V:G→[1,∞) let the transition kernel $M_{a}$ of the MH algorithm be V-uniformly ergodic, that is,

$${\left\|M_{a}^{n}\left(x,\cdot \right)-\pi \right\|}_{V}\leq CV\left(x\right)\alpha^{n}$$

with C∈[1,∞) and α∈[0,1), and additionally, assume that the Lyapunov condition

$$M_{a}V\left(x\right)\leq \delta V\left(x\right)+L,$$

for some δ∈[0,1) and L∈[1,∞) is satisfied.

We have the following standard proposition (see e.g. [26, Lemma 4.1] or [1], [4], [10], [15], [22]) which leads to upper bounds on $\varepsilon_{\mathrm{tv}}$, $\varepsilon_{V}$ and Δ(R) (see Lemma 4 and Theorem 9) for two MH type algorithms $M_{b}$ and $M_{c}$ with common proposal distribution but different acceptance probability functions b,c:G×G→[0,1], respectively.

Proposition 12 *Let* b,c:G×G→[0,1] *and let*V:G→[1,∞) *be such that*$\sup_{x\in G}\frac{M_{b}V\left(x\right)}{V\left(x\right)}\leq T$ *for a constant*T≥1*. Assume that there are functions* η,ξ:G→[0,∞) *and a set* B⊆G *such that, either*

(20) $$\left|b\left(x,y\right)-c\left(x,y\right)\right|\leq 1_{B}\left(y\right)\left(\eta \left(x\right)+\eta \left(y\right)\right)b\left(x,y\right)\xi \left(x\right),\;\text{or}\left|b\left(x,y\right)-c\left(x,y\right)\right|\leq 1_{B}\left(y\right)\left(\eta \left(x\right)+\eta \left(y\right)\right)b\left(x,y\right)\xi \left(y\right)$$

*for all* x,y∈G*. Then we have*

$$\underset{x\in B}{\sup}\frac{{\left\|M_{b}\left(x,\cdot \right)-M_{c}\left(x,\cdot \right)\right\|}_{V}}{V\left(x\right)}\leq 4T{\left\|\eta \cdot 1_{B}\right\|}_{\infty }{\left\|\xi \cdot 1_{B}\right\|}_{\infty },$$

*and, with the definition of* ${\left\|\cdot \right\|}_{\infty ,W}$ *provided in* (9)*, for any* β∈(0,1)*,*

$$\underset{x\in B}{\sup}\frac{{\left\|M_{b}\left(x,\cdot \right)-M_{c}\left(x,\cdot \right)\right\|}_{\mathrm{tv}}}{V\left(x\right)}\leq 4T{\left\|\eta \cdot 1_{B}\right\|}_{\infty ,V^{\beta }}{\left\|\xi \cdot 1_{B}\right\|}_{\infty ,V^{1-\beta }}.$$

The proposition provides a tool for controlling the distance between the transition kernels of two MH type algorithms with identical proposal and different acceptance probabilities. The specific functional form for the dependence of the upper bound in (20) on x and y is motivated by the applications below. The set B indicates the “essential” part of G where the difference of the acceptance probabilities matter. The parameter β is used to shift weight between the two components ξ and η of the approximation error. For the proof of the proposition, we refer to Appendix A.2.

### 4.1 Doubly-intractable distributions

In the case where $\pi_{u}$ takes the form (18), we can approximate Z(x) by a Monte Carlo estimate

**Figure:**
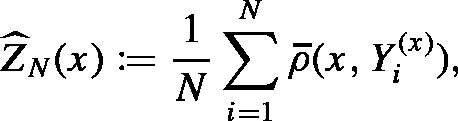

under the assumption that we have access to an iid sequence of random variables ${\left(Y_{i}^{\left(x\right)}\right)}_{1\leq i\leq N}$ where each $Y_{i}^{\left(x\right)}$ is distributed according to $r_{x}$. Then, the idea is to substitute the unknown quantity Z(x) by the approximation Z^N(x) within the acceptance ratio. Defining WN(x)≔Z^N(x)Z(x), the acceptance ratio can be written as

r˜(x,z,WN(x),WN(z))≔μ(dz)Q(z,dx)μ(dx)Q(x,dz)⋅Z^N(x)Z^N(z)=r(x,z)⋅WN(x)WN(z),

where the random variables $W_{N}\left(x\right)$, $W_{N}\left(z\right)$ are assumed to be independent from each other. Notice that the quantities $W_{N}$ only appear in the theoretical analysis of the algorithm. For the implementation, it is sufficient to be able to compute $\overset{˜}{r}$. This leads to a *Monte Carlo within Metropolis* (MCwM) algorithm:

Algorithm 2 For a given proposal transition kernel Q, a transition from x to y of the MCwM algorithm works as follows.

1. Draw U∼Unif[0,1] and a proposal Z∼Q(x,⋅) independently, call the result u and z, respectively.
2. Calculate $\overset{˜}{r}\left(x,z,W_{N}\left(x\right),W_{N}\left(z\right)\right)$ based on independent samples for $W_{N}\left(x\right)$, $W_{N}\left(z\right)$, which are also independent from previous iterations.
3. If $u<\overset{˜}{r}\left(x,z,W_{N}\left(x\right),W_{N}\left(z\right)\right)$, then accept the proposal, and return y≔z, otherwise reject the proposal and return y≔x.

Given the current state x∈G and a proposed state z∈G the overall acceptance probability is

(21) $$a_{N}\left(x,z\right)≔E\left[\min\left\{1,\overset{˜}{r}\left(x,z,W_{N}\left(x\right),W_{N}\left(z\right)\right)\right\}\right],$$

which leads to the corresponding transition kernel of the form $M_{a_{N}}$, see (17).

Remark 13 Let us emphasize that the doubly-intractable case can also be approached algorithmically from various other perspectives. For instance, instead of estimating the normalizing constant Z(x) one could estimate unbiasedly ${\left(Z\left(x\right)\right)}^{-1}$ whenever exact simulation from the Markov or Gibbs random field is possible. In this case, $\pi_{u}\left(x\right)$ turns into a Monte Carlo estimate which can formally be analyzed with exactly the same techniques as the latent variable scenario described below. Yet another algorithmic possibility is explored in the *noisy exchange* algorithm of [1], where ratios of the form Z(x)∕Z(y) are approximated by a single Monte Carlo estimate. Their algorithm is motivated by the *exchange algorithm* [19] which, perhaps surprisingly, can avoid the need for evaluating the ratio Z(x)∕Z(y) and targets the distribution π exactly, see e.g. [6], [21] for an overview of these and related methods. However, in some cases the exchange algorithm performs poorly, see [1]. Then approximate sampling methods for distributions of the form (2) might prove useful as long as the introduced bias is not too large. As a final remark in this direction, the recent work  [2] considers a correction of the noisy exchange algorithm which produces a Markov chain with stationary distribution π.

The quality of the MCwM algorithm depends on the error of the approximation of Z(x). The root mean squared error of this approximation can be quantified by the use of $W_{N}$, that is,

(22) $${\left(E{\left|W_{N}\left(x\right)-1\right|}^{2}\right)}^{1∕2}=\frac{s\left(x\right)}{\sqrt{N}}\;x\in G,\;N\in N,$$

where

$$s\left(x\right)≔{\left(E{\left|W_{1}\left(x\right)-1\right|}^{2}\right)}^{1∕2}$$

is determined by the second moment of $W_{1}\left(x\right)$. In addition, due to the appearance of the estimator $W_{N}\left(z\right)$ in the denominator of $\overset{˜}{r}$, we need some control of its distribution near zero. To this end, we define, for z∈G and p>0, the inverse moment function

$$i_{p,N}\left(z\right)≔{\left(EW_{N}{\left(z\right)}^{-p}\right)}^{\frac{1}{p}}.$$

With this notation we obtain the following estimate, which is proved in Appendix A.2.

Lemma 14 *Assume that there exists* k∈N *such that* $i_{2,k}\left(x\right)$ *and*s(x) *are finite for all* x∈G*. Then, for all* x,z∈G *and* N≥k *we have*

$$\left|a\left(x,z\right)-a_{N}\left(x,z\right)\right|\leq a\left(x,z\right)\frac{1}{\sqrt{N}}i_{2,k}\left(z\right)\left(s\left(x\right)+s\left(z\right)\right).$$

Remark 15 One can replace the boundedness of the second inverse moment $i_{2,k}\left(x\right)$ for any x∈G by boundedness of a lower moment $i_{p,m}\left(x\right)$ for p∈(0,2) with suitably adjusted m∈N, see Lemma 23 in the Appendix A.2.

#### 4.1.1 Inheritance of the Lyapunov condition

If the second and inverse second moment are uniformly bounded, ${\left\|s\right\|}_{\infty }<\infty$ as well as ${\left\|i_{2,N}\right\|}_{\infty }<\infty$, one can show that the Lyapunov condition of the MH transition kernel is inherited by the MCwM algorithm. In the following corollary, we prove this inheritance and state the resulting error bound for MCwM.

Corollary 16 *For a distribution* $m_{0}$ *on* G *let*$m_{n}≔m_{0}M_{a}^{n}$ *and*$m_{n,N}≔m_{0}M_{a_{N}}^{n}$ *be the respective distributions of the MH and MCwM algorithms after*n *steps. Let* Assumption 11 *be satisfied and for some* k∈N *let*

$$D≔8L{\left\|i_{2,k}\right\|}_{\infty }{\left\|s\right\|}_{\infty }<\infty .$$

*Further, define* $\delta_{N}≔\delta +D∕\sqrt{N}$ *and* $\beta_{n}≔n\max{\left\{\delta_{N},\alpha \right\}}^{n-1}.$ *Then, for any*

$$N>\max\left\{k,\frac{D^{2}}{{\left(1-\delta \right)}^{2}}\right\}$$

*we have* $\delta_{N}\in \left[0,1\right)$ *and*

$${\left\|m_{n}-m_{n,N}\right\|}_{\mathrm{tv}}\leq \frac{DC}{\sqrt{N}}\left[m_{0}\left(V\right)\beta_{n}+\frac{L}{\left(1-\delta_{N}\right)\left(1-\alpha \right)}\right].$$

Proof Assumption 11 implies $\sup_{x\in G}\frac{M_{a}V\left(x\right)}{V\left(x\right)}\leq 2L$. By Lemma 14 and Proposition 12, with B=G, we obtain

$$\varepsilon_{V,V}=\underset{x\in G}{\sup}\frac{{\left\|M_{a}\left(x,\cdot \right)-M_{a_{N}}\left(x,\cdot \right)\right\|}_{V}}{V\left(x\right)}\leq \frac{D}{\sqrt{N}}.$$

Further, note that

$$M_{a_{N}}V\left(x\right)-M_{a}V\left(x\right)\leq {\left\|M_{a}\left(x,\cdot \right)-M_{a_{N}}\left(x,\cdot \right)\right\|}_{V}\leq \frac{D}{\sqrt{N}}V\left(x\right),$$

which implies, by Assumption 11, that for $N>D^{2}∕{\left(1-\delta \right)}^{2}$ we have $\delta_{N}\in \left[0,1\right)$ and $M_{a_{N}}V\left(x\right)\leq \delta_{N}V\left(x\right)+L$. By Theorem 7 and Remark 8 we obtain for r=1 the assertion.  □

Observe that the estimate is bounded in n∈N so that the difference of the distributions converges uniformly in n to zero for N→∞. The constant $\delta_{N}$ decreases for increasing N, so that larger values of N improve the bound.

**Log-normal example I.** Let G=R and the target measure π be the standard normal distribution. We choose a Gaussian proposal kernel $Q\left(x,\cdot \right)=N\left(x,\gamma^{2}\right)$ for some $\gamma^{2}>0$, where $N\left(\mu ,\sigma^{2}\right)$ denotes the normal distribution with mean μ and variance $\sigma^{2}$. It is well known, see [9, Theorem 4.1, Theorem 4.3 and Theorem 4.6], that the MH transition kernel satisfies Assumption 11 for some numbers α, C, δ and L with $V\left(x\right)=\exp\left(x^{2}∕4\right)$.

Let $g\left(y;\mu ,\sigma^{2}\right)$ be the density of the log-normal distribution with parameters μ and σ, i.e., g is the density of exp(μ+σS) for a random variable S∼N(0,1). Then, by the fact that $\int_{0}^{\infty }y\;g\left(y;-\sigma {\left(x\right)}^{2}∕2,\sigma {\left(x\right)}^{2}\right)dy=1$ for all functions σ:G→(0,∞), we can write the (unnormalized) standard normal density as

$$\pi_{u}\left(x\right)=\exp\left(-x^{2}∕2\right)=\frac{\exp\left(-x^{2}∕2\right)}{\int_{0}^{\infty }y\;g\left(y;-\sigma {\left(x\right)}^{2}∕2,\sigma {\left(x\right)}^{2}\right)dy}.$$

Hence $\pi_{u}$ takes the form (18) with Y=[0,∞), $\rho \left(x\right)=\exp\left(-x^{2}∕2\right)$,  and $r_{x}$ being a log-normal distribution with parameters $-\sigma {\left(x\right)}^{2}∕2$ and $\sigma {\left(x\right)}^{2}$. Independent draws from this log-normal distribution are used in the MCwM algorithm to approximate the integral. We have $E\left[W_{1}{\left(x\right)}^{p}\right]=\exp\left(p\left(p-1\right)\sigma {\left(x\right)}^{2}∕2\right)$ for all x,p∈R and, accordingly,

$$s\left(x\right)={\left(\exp\left(\sigma {\left(x\right)}^{2}\right)-1\right)}^{1∕2}\leq \exp\left(\sigma {\left(x\right)}^{2}∕2\right)$$

$$i_{p,1}\left(x\right)=\exp\left(\left(p+1\right)\sigma {\left(x\right)}^{2}∕2\right).$$

By Lemma 23 we conclude that

$$i_{2,k}\left(x\right)\leq i_{2∕k,1}\left(x\right)=\exp\left(\left(\frac{1}{2}+\frac{1}{k}\right)\sigma {\left(x\right)}^{2}\right).$$

**Fig. 1:**
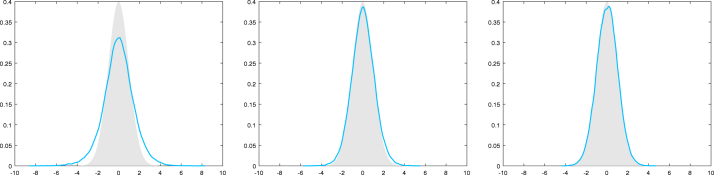
Here $\sigma {\left(x\right)}^{2}≔{\left|x\right|}^{1.8}$ for x∈R. The target density (standard normal) is plotted in gray, a kernel density estimator based on $10^{5}$ steps of the MCwM algorithm with N=10 (left), $N=10^{2}$ (middle) and $N=10^{3}$ (right) is plotted in blue . (For interpretation of the references to colour in this figure legend, the reader is referred to the web version of this article.)

Hence, ${\left\|s\right\|}_{\infty }$ as well as ${\left\|i_{2,k}\right\|}_{\infty }$ are bounded if for some constant c>0 we have $\sigma {\left(x\right)}^{2}\leq c$ for all x∈G. In that case Corollary 16 is applicable and provides estimates for the difference between the distributions of the MH and MCwM algorithms after n-steps. However, one might ask what happens if the function $\sigma {\left(x\right)}^{2}$ is not uniformly bounded, taking, for example, the form $\sigma {\left(x\right)}^{2}={\left|x\right|}^{q}$ for some q>0. In Fig. 1 we illustrate the difference of the distribution of the target measure to a kernel density estimator based on a MCwM algorithm sample for $\sigma {\left(x\right)}^{2}={\left|x\right|}^{1.8}$. Even though s(x) and $i_{p,1}\left(x\right)$ grow super-exponentially in |x|, the MCwM still works reasonably well in this case.

**Fig. 2:**
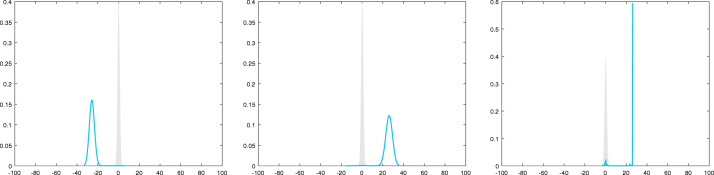
Here $\sigma {\left(x\right)}^{2}≔{\left|x\right|}^{2.2}$ for x∈R. The target density (standard normal) is plotted in gray, a kernel density estimator based on $10^{5}$ steps of the MCwM algorithm with N=10 (left), $N=10^{2}$ (middle) and $N=10^{3}$ (right) is plotted in blue . (For interpretation of the references to colour in this figure legend, the reader is referred to the web version of this article.)

However, in Fig. 2 we consider the case where $\sigma {\left(x\right)}^{2}={\left|x\right|}^{2.2}$ and the behavior changes dramatically. Here the MCwM algorithm does not seem to work at all. This motivates a modification of the MCwM algorithm in terms of restricting the state space to the “essential part” determined by the Lyapunov condition.

#### 4.1.2 Restricted MCwM approximation

With the notation and definition from the previous section we consider the case where the functions $i_{2,k}\left(x\right)$ and s(x) are not uniformly bounded. Under Assumption 11 there are two simultaneously used tools which help to control the difference of a transition of MH and MCwM:

1. The Lyapunov condition leads to a weight function and eventually to a weighted norm, see Proposition 12.
2. By restricting the MCwM to the “essential part” of the state space we prevent that the approximating Markov chain deteriorates. Namely, for some R≥1 we restrict the MCwM to $B_{R}$, see Section 3.2.

For x,z∈G the acceptance ratio $\overset{˜}{r}$ used in Algorithm 2 is now modified to

$$1_{B_{R}}\left(z\right)\cdot \overset{˜}{r}\left(x,z,W_{N}\left(x\right),W_{N}\left(z\right)\right)$$

which leads to the *restricted MCwM algorithm*:

Algorithm 3 For given R≥1 and a proposal transition kernel Q a transition from x to y of the restricted MCwM algorithm works as follows.

1. Draw U∼Unif[0,1] and a proposal Z∼Q(x,⋅) independently, call the result u and z, respectively.
2. Calculate $\overset{˜}{r}\left(x,z,W_{N}\left(x\right),W_{N}\left(z\right)\right)$ based on independent samples for $W_{N}\left(x\right)$, $W_{N}\left(z\right)$, which are also independent from previous iterations.
3. If $u<1_{B_{R}}\left(z\right)\cdot \overset{˜}{r}\left(x,z,W_{N}\left(x\right),W_{N}\left(z\right)\right)$, then accept the proposal, and return y≔z, otherwise reject the proposal and return y≔x.

Given the current state x∈G and a proposed state z∈G the overall acceptance probability is

$$a_{N}^{\left(R\right)}\left(x,z\right)≔E\left[\min\left\{1,1_{B_{R}}\left(z\right)\cdot \overset{˜}{r}\left(x,z,W_{N}\left(x\right),W_{N}\left(z\right)\right)\right\}\right]=1_{B_{R}}\left(z\right)\cdot a_{N}\left(x,z\right),$$

which leads to the corresponding transition kernel of the form $M_{a_{N}^{\left(R\right)}}$, see (17). By using Theorem 9 and Proposition 12 we obtain the following estimate.

Corollary 17 *Let* Assumption 11 *be satisfied, i.e.,* $M_{a}$ *is* V*-uniformly ergodic and the function* V *as well as the constants* α,C,δ *and* L *are determined. For* β∈(0,1) *and* R≥1 *let*

$$B_{R}≔\left\{x\in G|V\left(x\right)\leq R\right\},$$

$$D_{R}≔12\cdot L{\left\|i_{2,k}\cdot 1_{B_{R}}\right\|}_{\infty ,V^{1-\beta }}{\left\|s\cdot 1_{B_{R}}\right\|}_{\infty ,V^{\beta }}<\infty .$$

*Let* $m_{0}$ *be a distribution on* $B_{R}$ *and* $\kappa ≔\max\left\{m_{0}\left(V\right),L∕\left(1-\delta \right)\right\}$*. Then, for*

(23) $$N\geq \max\left\{k,4{\left(\frac{R\cdot D_{R}}{1-\delta }\right)}^{2}\right\}$$

*and* R≥exp(1) *we have*

$${\left\|m_{n}-m_{n,N}^{\left(R\right)}\right\|}_{\mathrm{tv}}\leq \frac{33C\left(L+1\right)\kappa }{1-\alpha }\cdot \frac{\log R}{R},$$

*where* $m_{n,N}^{\left(R\right)}≔m_{0}M_{a_{N}^{\left(R\right)}}^{n}$ *and* $m_{n}≔m_{0}M_{a}^{n}$ *are the distributions of the MH and restricted MCwM algorithm after*n*-steps.*

Proof We apply Theorem 9 with $P\left(x,\cdot \right)=M_{a}\left(x,\cdot \right)$ and

$$\overset{˜}{P}\left(x,\cdot \right)=1_{B_{R}}\left(x\right)\;M_{a_{N}^{\left(R\right)}}\left(x,\cdot \right)+1_{B_{R}^{c}}\left(x\right)\delta_{x_{0}}\left(\cdot \right),\;x\in G,$$

for some $x_{0}\in B_{R}$. Note that $\overset{˜}{P}\left(x,B_{R}\right)=1$ for any x∈G. Further $\overset{˜}{P}$ and $M_{a_{N}^{\left(R\right)}}$ coincide on $B_{R}$, thus we also have ${\overset{˜}{P}}^{n}=M_{a_{N}^{\left(R\right)}}^{n}$ on $B_{R}$ for n∈N. Observe also that the restriction of P to $B_{R}$, denoted by $P_{R}$, satisfies $P_{R}=M_{a^{\left(R\right)}}$ with $a^{\left(R\right)}\left(x,z\right)≔1_{B_{R}}\left(z\right)\;a\left(x,z\right)$. Hence

$$\Delta \left(R\right)=\underset{x\in B_{R}}{\sup}\frac{{\left\|M_{a^{\left(R\right)}}\left(x,\cdot \right)-M_{a_{N}^{\left(R\right)}}\left(x,\cdot \right)\right\|}_{\mathrm{tv}}}{V\left(x\right)}.$$

Moreover, we have by Lemma 14 that

$$\left|a^{\left(R\right)}\left(x,z\right)-a_{N}^{\left(R\right)}\left(x,z\right)\right|=1_{B_{R}}\left(z\right)\left|a\left(x,z\right)-a_{N}\left(x,z\right)\right|\leq 1_{B_{R}}\left(z\right)\cdot a\left(x,z\right)\frac{1}{\sqrt{N}}\;i_{2,k}\left(z\right)\left(s\left(x\right)+s\left(z\right)\right)=a^{\left(R\right)}\left(x,z\right)\frac{1}{\sqrt{N}}\;i_{2,k}\left(z\right)\left(s\left(x\right)+s\left(z\right)\right).$$

With Proposition 12 and

$$\underset{x\in G}{\sup}\frac{M_{a^{\left(R\right)}}V\left(x\right)}{V\left(x\right)}\leq \underset{x\in G}{\sup}\frac{M_{a}V\left(x\right)}{V\left(x\right)}+1\underset{\text{Assumption 11}}{\leq }3L,$$

we have that $\Delta \left(R\right)\leq D_{R}∕\sqrt{N}$. Then, by $N\geq 4{\left(RD_{R}∕\left(1-\delta \right)\right)}^{2}$ we obtain

$$R\cdot \Delta \left(R\right)\leq \frac{1-\delta }{2}$$

such that all conditions of Theorem 9 are verified and the stated estimate follows. □

Remark 18 The estimate depends crucially on the sample size N as well as on the parameter R. If the influence of R in $D_{R}$ is explicitly known, then one can choose R depending on N in such away that the conditions of the corollary are satisfied and one eventually obtains an upper bound on the total variation distance of the difference between the distributions depending only on N and not on R anymore. For example, if we additionally assume that the function g:(0,∞)→(0,∞) given by $g\left(R\right)=R\cdot D_{R}$ is invertible, then for N≥k and the choice $R≔g^{-1}\left(\left(1-\delta \right)\sqrt{N}∕2\right)$ we have

$${\left\|m_{n}-m_{n,N}^{\left(R\right)}\right\|}_{\mathrm{tv}}\leq \frac{33C\left(L+1\right)\kappa }{1-\alpha }\cdot \frac{\log\left(g^{-1}\left(\left(1-\delta \right)\sqrt{N}∕2\right)\right)}{g^{-1}\left(\left(1-\delta \right)\sqrt{N}∕2\right)}.$$

Thus, depending on whether and how fast $g^{-1}\left(\left(1-\delta \right)\sqrt{N}∕2\right)\to \infty$ for N→∞ determines the convergence of the upper bound of ${\left\|m_{n}-m_{n,N}^{\left(R\right)}\right\|}_{\mathrm{tv}}$ to zero.

**Log-normal example II.** We continue with the log-normal example. In this setting we have

$$B_{R}=\left\{x\in R|\left|x\right|\leq 2\sqrt{\log R}\right\},{\left\|i_{2,k}\cdot 1_{B_{R}}\right\|}_{\infty ,V^{1-\beta }}\leq \underset{\left|x\right|\leq 2\sqrt{\log R}}{\sup}\exp\left(\left(\frac{1}{2}+\frac{1}{k}\right)\sigma {\left(x\right)}^{2}-\frac{1-\beta }{4}x^{2}\right),{\left\|s\cdot 1_{B_{R}}\right\|}_{\infty ,V^{\beta }}\leq \underset{\left|x\right|\leq 2\sqrt{\log R}}{\sup}\exp\left(\sigma {\left(x\right)}^{2}∕2-\beta x^{2}∕4\right).$$

**Fig. 3:**
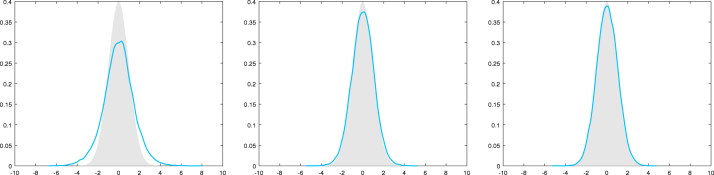
Here $\sigma {\left(x\right)}^{2}≔{\left|x\right|}^{1.8}$ for x∈R and $B_{R}=\left[-10,10\right]$. The target density (standard normal) is plotted in gray, a kernel density estimator based on $10^{5}$ steps of the MCwM algorithm with N=10 (left), $N=10^{2}$ (middle) and $N=10^{3}$ (right) is plotted in blue.

**Fig. 4:**
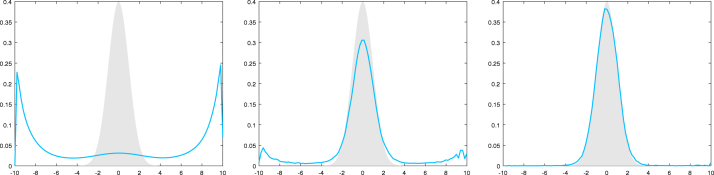
Here $\sigma {\left(x\right)}^{2}≔{\left|x\right|}^{2.2}$ for x∈R and $B_{R}=\left[-10,10\right]$. The target density (standard normal) is plotted in gray, a kernel density estimator based on $10^{5}$ steps of the MCwM algorithm with N=10 (left), $N=10^{2}$ (middle) and $N=10^{3}$ (right) is plotted in blue.

Thus, $D_{R}$ is uniformly bounded in R for $\sigma {\left(x\right)}^{2}\propto {\left|x\right|}^{q}$ with q<2 and not uniformly bounded for q>2. As in the numerical experiments in Fig. 1, Fig. 2 let us consider the cases $\sigma {\left(x\right)}^{2}={\left|x\right|}^{1.8}$ and $\sigma {\left(x\right)}^{2}={\left|x\right|}^{2.2}$. In Fig. 3 we compare the normal target density with a kernel density estimator based on the restricted MCwM on $B_{R}=\left[-10,10\right]$ and observe essentially the same reasonable behavior as in Fig. 1. In Fig. 4 we consider the same scenario and observe that the restriction indeed stabilizes. In contrast to Fig. 2, convergence to the true target distribution is visible but, in line with the theory, slower than for $\sigma {\left(x\right)}^{2}={\left|x\right|}^{1.8}$.

 Now we apply Corollary 17 in both cases and note that by similar arguments as below one can also treat $\sigma {\left(x\right)}^{2}\propto {\left|x\right|}^{q}$ with, respectively, q<2 or q>2.

**1. Case** $\sigma {\left(x\right)}^{2}={\left|x\right|}^{1.8}$. For k=100 and β=1∕2 one can easily see that ${\left\|i_{2,100}\cdot 1_{B_{R}}\right\|}_{\infty ,V^{1∕2}}$ and ${\left\|s\cdot 1_{B_{R}}\right\|}_{\infty ,V^{1∕2}}$ is bounded by 6000, independent of R. Hence there is a constant D≥1 so that $D_{R}\leq D$. With this knowledge we choose $R=\frac{\left(1-\delta \right)}{\sqrt{2}D}\sqrt{N}$ such that for $N\geq \max\left\{100,\frac{2\exp\left(2\right)D^{2}}{{\left(1-\delta \right)}^{2}}\right\}$ condition (23) and R≥exp(1) is satisfied. Then, Corollary 17 gives the existence of a constant $\overset{˜}{C}>0$, so that

$${\left\|m_{n}-m_{n,N}^{\left(R\right)}\right\|}_{\mathrm{tv}}\leq \overset{˜}{C}\;\frac{\log N}{\sqrt{N}}$$

for any initial distribution $m_{0}$ on $B_{R}$.

**2. Case** $\sigma {\left(x\right)}^{2}={\left|x\right|}^{2.2}$. For k=100 and β=1∕2 we obtain

$${\left\|i_{2,100}\cdot 1_{B_{R}}\right\|}_{\infty ,V^{1∕2}}\leq \exp\left(2.5{\left(\log R\right)}^{11∕10}\right),{\left\|s\cdot 1_{B_{R}}\right\|}_{\infty ,V^{1∕2}}\leq \exp\left(2.5{\left(\log R\right)}^{11∕10}\right).$$

Hence $D_{R}\leq 12L\exp\left(5{\left(\log R\right)}^{11∕10}\right).$ Eventually, for

$$N\geq \max\left\{100,\frac{24^{2}\exp\left(2\cdot 6^{11∕10}\right)L^{2}}{{\left(1-\delta \right)}^{2}}\right\}$$

we have with $R=\exp\left(\frac{1}{6}{\left[\log\left(\frac{\sqrt{N}\left(1-\delta \right)}{24L}\right)\right]}^{10∕11}\right)$ that R≥exp(1) and (23) is satisfied. Then, with ${\overset{˜}{C}}_{1}≔\frac{33C\left(L+1\right)\kappa }{1-\alpha }$, ${\overset{˜}{C}}_{2}≔\sqrt{\frac{1-\delta }{24L}}$ and Corollary 17 we have

$${\left\|m_{n}-m_{n,N}^{\left(R\right)}\right\|}_{\mathrm{tv}}\leq \frac{{\overset{˜}{C}}_{1}\cdot \frac{1}{6\cdot 2^{10∕11}}{\left[\log\left({\overset{˜}{C}}_{2}N\right)\right]}^{10∕11}}{\exp\left(\frac{1}{6\cdot 2^{10∕11}}{\left[\log\left({\overset{˜}{C}}_{2}N\right)\right]}^{10∕11}\right)}\leq \frac{{\overset{˜}{C}}_{1}\left(k+1\right)!}{{\left[\log\left({\overset{˜}{C}}_{2}N\right)\right]}^{10k∕11}},\;$$

for any initial distribution $m_{0}$ on $B_{R}$ and all k∈N. Here the last inequality follows by the fact that $\exp\left(x\right)\geq \frac{x^{k+1}}{\left(k+1\right)!}$ for any x≥0 and k∈N.

To summarize, by suitably choosing N and R (possibly depending on N) sufficiently large the difference between the distributions of the restricted MCwM and the MH algorithms after n-steps can be made arbitrarily small.

### 4.2 Latent variables

In this section we consider $\pi_{u}$ of the form (19). Here, as for doubly intractable distributions, the idea is to substitute $\pi_{u}\left(x\right)$ in the acceptance probability of the MH algorithm by a Monte Carlo estimate

**Figure:**
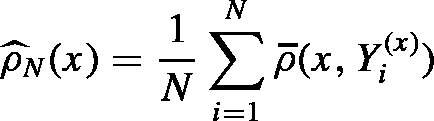

where we assume that we have access to an iid sequence of random variables ${\left(Y_{i}^{\left(x\right)}\right)}_{1\leq i\leq N}$ where each $Y_{i}^{\left(x\right)}$ has distribution $r_{x}$. Define a function $W_{N}:G\to R$ by WN(x)≔ρ^N(x)∕πu(x) and note that $E\left[W_{N}\left(x\right)\right]=1$. Then, the acceptance probability given $W_{N}\left(x\right)$, $W_{N}\left(z\right)$ modifies to

$$a_{N}\left(x,z\right)≔E\left[\min\left\{1,r\left(x,z\right)\cdot \frac{W_{N}\left(z\right)}{W_{N}\left(x\right)}\right\}\right]$$

where $W_{N}\left(x\right)$, $W_{N}\left(z\right)$ are assumed to be independent random variables. Note that all the objects which depend on $a_{N}$, such as $M_{a_{N}},a_{N}^{\left(R\right)},M_{a_{N}^{\left(R\right)}}$, that appear in this section are defined just as in Section 4.1. The only difference is that the order of the variables $W_{N}\left(x\right)$ and $W_{N}\left(z\right)$ in the ratio $\overset{˜}{r}$ at (21) has been reversed. Thus, this leads to a MCwM algorithm as stated in Algorithm 2, where the transition kernel is given by $M_{a_{N}}$.

Also as in Section 4.1 we define $s\left(x\right)≔{\left(E{\left|W_{1}\left(x\right)-1\right|}^{2}\right)}^{1∕2}$ and $i_{p,N}\left(x\right)≔{\left(EW_{N}{\left(x\right)}^{-p}\right)}^{1∕p}$ for all x∈G and p>0. With those quantities we obtain the following estimate of the difference of the acceptance probabilities of $M_{a}$ and $M_{a_{N}}$ proved in Appendix A.2.

Lemma 19 *Assume that there exists* k∈N *such that* $i_{2,k}\left(x\right)$ *and*s(x) *are finite for all* x∈G*. Then, for all* x,z∈G *and* N≥k *we have*

(24) $$\left|a\left(x,z\right)-a_{N}\left(x,z\right)\right|\leq a\left(x,z\right)\;\frac{1}{\sqrt{N}}\;i_{2,k}\left(x\right)\left(s\left(x\right)+s\left(z\right)\right).$$

If ${\left\|s\right\|}_{\infty }$ and ${\left\|i_{2,k}\right\|}_{\infty }$ are finite for some k∈N, then the same statement as formulated in Corollary 16 holds. The proof works exactly as stated there. Examples which satisfy this condition are for instance presented in [15]. However, there are cases where the functions s and $i_{2,k}$ are unbounded. In this setting, as in Section 4.1.2, we consider the restricted MCwM algorithm with transition kernel $M_{a_{N}^{\left(R\right)}}$. Here again the same statement and proof as formulated in Corollary 17 hold. We next provide an application of this corollary in the latent variable setting.

**Normal–normal model.** Let G=R and the function $\varphi_{\mu ,\sigma^{2}}$ be the density of $N\left(\mu ,\sigma^{2}\right)$. For some z∈R and (precision) parameters $\gamma_{Z},\gamma_{Y}>0$ define

$$\pi_{u}\left(x\right)≔\int_{R}\varphi_{z,\gamma_{Z}^{-1}}\left(y\right)\;\varphi_{0,\gamma_{Y}^{-1}}\left(x-y\right)dy,$$

that is, Y=R,  and $r_{x}=N\left(x,\gamma_{Y}^{-1}\right)$. By the convolution of two normals the target distribution π satisfies

(25) $$\pi_{u}\left(x\right)=\varphi_{z,\gamma_{Z,Y}^{-1}}\left(x\right),\;\text{with}\;\gamma_{Z,Y}^{-1}≔\gamma_{Z}^{-1}+\gamma_{Y}^{-1}.$$

Note that, for real-valued random variables Y,Z the probability measure π is the posterior distribution given an observation Z=z within the model

$$Z\left|Y=y∼N\left(y,\gamma_{Z}^{-1}\right),\;Y\right|x∼N\left(x,\gamma_{Y}^{-1}\right),$$

with the improper Lebesgue prior imposed on x.

Pretending that we do not know $\pi_{u}\left(x\right)$ we compute

ρ^N(x)=1N∑i=1Nφz,γZ−1(Yi(x)),

where ${\left(Y_{i}^{\left(x\right)}\right)}_{1\leq i\leq N}$ is a sequence of iid random variables with $Y_{1}^{\left(x\right)}∼N\left(x,\gamma_{Y}^{-1}\right)$. Hence

$$W_{N}\left(x\right)=\frac{1}{N}\overset{N}{\underset{i=1}{\sum }}\frac{\varphi_{z,\gamma_{Z}^{-1}}\left(Y_{i}^{\left(x\right)}\right)}{\varphi_{z,\gamma_{Z,Y}^{-1}}\left(x\right)}=\frac{1}{N}{\left(\frac{\gamma_{Z}}{\gamma_{Z,Y}}\right)}^{1∕2}\overset{N}{\underset{i=1}{\sum }}\frac{\varphi_{0,1}\left(\sqrt{\gamma_{Z}}\left(z-Y_{i}^{\left(x\right)}\right)\right)}{\varphi_{0,1}\left(\sqrt{\gamma_{Z,Y}}\left(z-x\right)\right)}.$$

By using a random variable ξ∼N(0,1) we have for $p>-\gamma_{Y}∕\gamma_{Z}$ that

(26) $$E\left[W_{1}{\left(x\right)}^{p}\right]={\left(\frac{\gamma_{Z}}{\gamma_{Z,Y}}\right)}^{p∕2}E\left[\exp\left(\frac{p}{2}\gamma_{Z,Y}{\left(z-x\right)}^{2}-\frac{p}{2}\;\frac{\gamma_{Z}}{\gamma_{Y}}{\left(\gamma_{Y}^{1∕2}\left(z-x\right)-\xi \right)}^{2}\right)\right]\propto \exp\left(\frac{\gamma_{Z}\;\gamma_{Z,Y}\;p\left(p-1\right)}{2\left(\gamma_{Y}+p\gamma_{Z}\right)}{\left(z-x\right)}^{2}\right).$$

Here ∝ means equal up to a constant independent of x. As a consequence, ${\left\|s\right\|}_{\infty }=\infty$ and therefore Corollary 16 (which is also true in the latent variable setting) cannot be applied. Nevertheless, we can obtain bounds for the restricted MCwM in this example using the statement of Corollary 17 by controlling s and $i_{2,k}$ using a Lyapunov function V. The following result, proved in Appendix A.2, verifies the necessary moment conditions under some additional restrictions on the model parameters.

Proposition 20 *Assume that* $\gamma_{Y}>\sqrt{2}\gamma_{Z}$*, the unnormalized density* $\pi_{u}$ *is given as in* (25) *and let the proposal transition kernel*Q *be a Gaussian random walk, that is,* $Q\left(x,\cdot \right)=N\left(x,\sigma^{2}\right)$ *for some* σ>0*. Then, there is a Lyapunov function* V:G→[1,∞) *for* $M_{a}$*, such that* $M_{a}$ *is*V*-uniformly ergodic, i.e.,* Assumption 11 *is satisfied, and there are* β∈(0,1) *as well as* k∈N *such that*

$${\left\|i_{2,k}\right\|}_{\infty ,V^{1-\beta }}<\infty \;\text{and}\;{\left\|s\right\|}_{\infty ,V^{\beta }}<\infty .$$

The previous proposition implies that there is a constant D<∞, such that $D_{R}$ from Corollary 17 is bounded by D independent of R. Hence there are numbers ${\overset{˜}{C}}_{1},{\overset{˜}{C}}_{2}>0$ such that with $R={\overset{˜}{C}}_{1}\sqrt{N}$ and for N sufficiently large we have

$${\left\|m_{n}-m_{n,N}^{\left(R\right)}\right\|}_{\mathrm{tv}}\leq {\overset{˜}{C}}_{2}\;\frac{\log N}{\sqrt{N}}$$

for any initial distribution $m_{0}$ on $B_{R}$.

## Appendix Technical proofs

### A.1 Proofs of Section 3

Before we come to the proofs of Section 3 let us recall a relation between geometric ergodicity and an ergodicity coefficient. Let V:G→[1,∞] be a measurable, π-a.e. finite function, then, define the *ergodicity coefficient* $\tau_{V}\left(P\right)$ as

$$\tau_{V}\left(P\right)≔\underset{x,y\in G}{\sup}\frac{{\left\|P\left(x,\cdot \right)-P\left(y,\cdot \right)\right\|}_{V}}{V\left(x\right)+V\left(y\right)}.$$

The next lemma provides a relation between the ergodicity coefficient and V-uniform ergodicity.

Lemma 21 *If* (7) *is satisfied, then* $\tau_{V}\left(P^{n}\right)\leq C\alpha^{n}$*.*

A proof of this fact is implicitly contained in [13] and can also be found in [26, Lemma 3.2]. Both references crucially use an observation of Hairer and Mattingly [8].

To summarize, if the transition kernel P is geometrically ergodic, then, by Theorem 1 there exist a function V:G→[1,∞), α∈[0,1) and C∈(0,∞) such that, by Lemma 21, $\tau_{V}\left(P^{n}\right)\leq C\alpha^{n}$. The next proposition states two further useful properties (submultiplicativity and contractivity) of the ergodicity coefficient. For a proof of the corresponding inequalities see for example [13, Proposition 2.1].

Proposition 22 *Assume* P,Q *are transition kernels and* μ,ν *are probability measures on* G*. Then*

$$\tau_{V}\left(PQ\right)\leq \tau_{V}\left(P\right)\;\tau_{V}\left(Q\right),\;\text{(submultiplicativity)}{\left\|\left(\mu -\nu \right)P\right\|}_{V}\leq \tau_{V}\left(P\right){\left\|\mu -\nu \right\|}_{V}.\;\text{(contractivity)}$$

Now we prove Lemma 4.

Proof of Lemma 4 As in the proof of [18, Theorem 3.1] we use

$${\overset{˜}{p}}_{n}-p_{n}=\left({\overset{˜}{p}}_{0}-p_{0}\right)P^{n}+\overset{n-1}{\underset{i=0}{\sum }}{\overset{˜}{p}}_{i}\left(\overset{˜}{P}-P\right)P^{n-i-1},$$

which can be shown by induction over n∈N. Then

(A.1) $${\left\|{\overset{˜}{p}}_{n}-p_{n}\right\|}_{\mathrm{tv}}\leq {\left\|\left({\overset{˜}{p}}_{0}-p_{0}\right)P^{n}\right\|}_{\mathrm{tv}}+\overset{n-1}{\underset{i=0}{\sum }}{\left\|{\overset{˜}{p}}_{i}\left(\overset{˜}{P}-P\right)P^{n-i-1}\right\|}_{\mathrm{tv}}.$$

With Proposition 22 and Lemma 21 we estimate the first term of the previous inequality by

$${\left\|\left({\overset{˜}{p}}_{0}-p_{0}\right)P^{n}\right\|}_{\mathrm{tv}}\leq {\left\|\left({\overset{˜}{p}}_{0}-p_{0}\right)P^{n}\right\|}_{V}\leq \tau_{V}\left(P^{n}\right){\left\|{\overset{˜}{p}}_{0}-p_{0}\right\|}_{V}\leq C\alpha^{n}{\left\|{\overset{˜}{p}}_{0}-p_{0}\right\|}_{V}.$$

For the terms which appear in the sum of (A.1) we can use two types of estimates. Note that $\tau_{1}\left(P\right)\leq 1$ (here the subscript indicates that V=1) which leads by Proposition 22 to

$${\left\|{\overset{˜}{p}}_{i}\left(\overset{˜}{P}-P\right)P^{n-i-1}\right\|}_{\mathrm{tv}}\leq {\left\|{\overset{˜}{p}}_{i}\left(\overset{˜}{P}-P\right)\right\|}_{\mathrm{tv}}\tau_{1}\left(P^{n-i-1}\right)\leq {\left\|{\overset{˜}{p}}_{i}\left(\overset{˜}{P}-P\right)\right\|}_{\mathrm{tv}}=\underset{\left|f\right|\leq 1}{\sup}\left|\int_{G}f\left(x\right)\;{\overset{˜}{p}}_{i}\left(\overset{˜}{P}-P\right)\left(dx\right)\right|=\underset{\left|f\right|\leq 1}{\sup}\left|\int_{G}\left(\overset{˜}{P}-P\right)f\left(x\right)\;{\overset{˜}{p}}_{i}\left(dx\right)\right|\leq \int_{G}{\left\|\overset{˜}{P}\left(x,\cdot \right)-P\left(x,\cdot \right)\right\|}_{\mathrm{tv}}\;{\overset{˜}{p}}_{i}\left(dx\right)\leq \varepsilon_{\mathrm{tv},W}\;{\overset{˜}{p}}_{i}\left(W\right).$$

On the other hand

$${\left\|{\overset{˜}{p}}_{i}\left(\overset{˜}{P}-P\right)P^{n-i-1}\right\|}_{\mathrm{tv}}\leq {\left\|{\overset{˜}{p}}_{i}\left(\overset{˜}{P}-P\right)P^{n-i-1}\right\|}_{V}\leq {\left\|{\overset{˜}{p}}_{i}\left(\overset{˜}{P}-P\right)\right\|}_{V}\tau_{V}\left(P^{n-i-1}\right)\leq C\alpha^{n-i-1}{\left\|{\overset{˜}{p}}_{i}\left(\overset{˜}{P}-P\right)\right\|}_{V}\leq C\alpha^{n-i-1}\int_{G}{\left\|\overset{˜}{P}\left(x,\cdot \right)-P\left(x,\cdot \right)\right\|}_{V}{\overset{˜}{p}}_{i}\left(dx\right)\leq C\alpha^{n-i-1}\varepsilon_{V,W}\;{\overset{˜}{p}}_{i}\left(W\right).$$

Thus, for any r∈(0,1] we obtain

$${\left\|{\overset{˜}{p}}_{i}\left(\overset{˜}{P}-P\right)P^{n-i-1}\right\|}_{\mathrm{tv}}\leq {\left\|{\overset{˜}{p}}_{i}\left(\overset{˜}{P}-P\right)P^{n-i-1}\right\|}_{\mathrm{tv}}^{1-r}\cdot {\left\|{\overset{˜}{p}}_{i}\left(\overset{˜}{P}-P\right)P^{n-i-1}\right\|}_{\mathrm{tv}}^{r}\leq \varepsilon_{\mathrm{tv},W}^{1-r}\;\varepsilon_{V,W}^{r}\;C^{r}\;{\overset{˜}{p}}_{i}\left(W\right)\;\alpha^{\left(n-i-1\right)r},$$

which gives by (A.1) the final estimate. □

Next we prove Theorem 9.

Proof Theorem 9 Locally for $x\in B_{R}$ we have $P_{R}V\left(x\right)\leq PV\left(x\right)\leq \delta V\left(x\right)+L,$ and, eventually,

(A.2) $$\overset{˜}{P}V\left(x\right)\leq P_{R}V\left(x\right)+\left|\overset{˜}{P}V\left(x\right)-P_{R}V\left(x\right)\right|\leq \delta V\left(x\right)+R{\left\|\overset{˜}{P}\left(x,\cdot \right)-P_{R}\left(x,\cdot \right)\right\|}_{\mathrm{tv}}+L\leq \left(\delta +R\cdot \Delta \left(R\right)\right)V\left(x\right)+L.$$

We write $B_{R}^{c}$ for $G\setminus B_{R}$ and obtain for $x\in B_{R}^{c}$ that

(A.3) $$\overset{˜}{P}V\left(x\right)=\int_{B_{R}}V\left(y\right)\overset{˜}{P}\left(x,dy\right)\leq V\left(x\right).$$

Denote $\overset{˜}{\delta }≔\delta +R\cdot \Delta \left(R\right)\leq 1∕2+\delta ∕2<1$. For i≥2 we obtain by (A.2), (A.3) and $\left(1-{\overset{˜}{\delta }}^{i}\right)\leq 2\left(1-{\overset{˜}{\delta }}^{i-1}\right)$ that

$${\overset{˜}{p}}_{i}\left(V\right)\leq {\overset{˜}{\delta }}^{i}\;\int_{B_{R}}V\left(x\right)p_{0}\left(dx\right)+\left(1-{\overset{˜}{\delta }}^{i}\right)\frac{L}{1-\overset{˜}{\delta }}\;+{\overset{˜}{\delta }}^{i-1}\int_{B_{R}^{c}}\overset{˜}{P}V\left(x\right)p_{0}\left(dx\right)+\left(1-{\overset{˜}{\delta }}^{i-1}\right)\frac{L}{1-\overset{˜}{\delta }}\leq {\overset{˜}{\delta }}^{i-1}p_{0}\left(V\right)+\left(1-{\overset{˜}{\delta }}^{i-1}\right)\frac{3L}{1-\overset{˜}{\delta }}\leq 6\kappa .$$

Furthermore, $p_{0}\left(V\right)\leq \kappa$ and ${\overset{˜}{p}}_{1}\left(V\right)\leq 2\kappa$. Now it is easily seen that

$$\overset{n-1}{\underset{i=0}{\sum }}{\overset{˜}{p}}_{i}\left(V\right)\alpha^{\left(n-i-1\right)r}\leq \frac{6\kappa }{r\left(1-\alpha \right)}.$$

For $\varepsilon_{\mathrm{tv},V}$ we have

$$\varepsilon_{\mathrm{tv},V}\leq \max\left\{\underset{x\in B_{R}}{\sup}\frac{{\left\|P\left(x,\cdot \right)-\overset{˜}{P}\left(x,\cdot \right)\right\|}_{\mathrm{tv}}}{V\left(x\right)},\underset{x\in \overset{c}{\underset{R}{B}}}{\sup}\frac{{\left\|P\left(x,\cdot \right)-\overset{˜}{P}\left(x,\cdot \right)\right\|}_{\mathrm{tv}}}{V\left(x\right)}\right\}.$$

The second term in the maximum is bounded by 2∕R. For $x\in B_{R}$ we have

$${\left\|P\left(x,\cdot \right)-\overset{˜}{P}\left(x,\cdot \right)\right\|}_{\mathrm{tv}}\leq {\left\|P\left(x,\cdot \right)-P_{R}\left(x,\cdot \right)\right\|}_{\mathrm{tv}}+{\left\|P_{R}\left(x,\cdot \right)-\overset{˜}{P}\left(x,\cdot \right)\right\|}_{\mathrm{tv}}\leq 2P\left(x,B_{R}^{c}\right)+{\left\|P_{R}\left(x,\cdot \right)-\overset{˜}{P}\left(x,\cdot \right)\right\|}_{\mathrm{tv}}$$

so that the first term in the maximum satisfies

$$\underset{x\in B_{R}}{\sup}\frac{{\left\|P\left(x,\cdot \right)-\overset{˜}{P}\left(x,\cdot \right)\right\|}_{\mathrm{tv}}}{V\left(x\right)}\leq \Delta \left(R\right)+2\underset{x\in B_{R}}{\sup}\frac{P\left(x,B_{R}^{c}\right)}{V\left(x\right)}.$$

Consider a random variable $X_{1}^{x}$ with distribution P(x,⋅), $x\in B_{R}$. Applying Markov’s inequality to the random variable $V\left(X_{1}^{x}\right)$ leads to

$$PV\left(x\right)=E\left[V\left(X_{1}^{x}\right)\right]\geq R\cdot P\left(V\left(X_{1}^{x}\right)>R\right)=R\cdot P\left(x,B_{R}^{c}\right),$$

and thus

$$\underset{x\in B_{R}}{\sup}\frac{P\left(x,B_{R}^{c}\right)}{V\left(x\right)}\leq \underset{x\in B_{R}}{\sup}\frac{PV\left(x\right)}{R\cdot V\left(x\right)}\leq \frac{\delta +L}{R}.$$

Finally, R⋅Δ(R)<1−δ and L≥1 imply $\varepsilon_{\mathrm{tv},V}\leq \frac{2\left(L+1\right)}{R}.$ We obtain $\varepsilon_{V,V}\leq 2\left(L+1\right)$ by the use of

$${\left\|P\left(x,\cdot \right)-\overset{˜}{P}\left(x,\cdot \right)\right\|}_{V}\leq PV\left(x\right)+\overset{˜}{P}V\left(x\right),$$

the fact that $\sup_{x\in G}\frac{PV\left(x\right)}{V\left(x\right)}\leq \delta +L$ and

$$\underset{x\in G}{\sup}\frac{\overset{˜}{P}V\left(x\right)}{V\left(x\right)}\leq \max\left\{\underset{x\in B_{R}}{\sup}\frac{\overset{˜}{P}V\left(x\right)}{V\left(x\right)},\underset{x\in \overset{c}{\underset{R}{B}}}{\sup}\frac{\overset{˜}{P}V\left(x\right)}{V\left(x\right)}\right\}\underset{\text{(A.2), (A.3)}}{\leq }\max\left\{\overset{˜}{\delta }+L,1\right\}\leq L+1.$$

Then, by Lemma 4 for r∈(0,1],

$${\left\|p_{n}-{\overset{˜}{p}}_{n}\right\|}_{\mathrm{tv}}\leq \frac{12C^{r}\left(L+1\right)\kappa }{r\cdot R^{1-r}\left(1-\alpha \right)}\leq \frac{12C\left(L+1\right)\kappa }{r\cdot R^{1-r}\left(1-\alpha \right)}.$$

By minimizing over r we obtain for R≥exp(1) that

$${\left\|p_{n}-{\overset{˜}{p}}_{n}\right\|}_{\mathrm{tv}}\leq \frac{12C\left(L+1\right)\kappa }{1-\alpha }\cdot \frac{R^{1∕\log\left(R\right)}\log\left(R\right)}{R}.$$

Finally by the fact that $R^{1∕\log R}=\exp\left(1\right)<33∕12$ the assertion follows. □

### A.2 Proofs of Section 4

We start with the proof of Proposition 12.

Proof of Proposition 12 For any f:G→R we have

$$M_{b}f\left(x\right)-M_{c}f\left(x\right)=\int_{G}f\left(y\right)\left(b\left(x,y\right)-c\left(x,y\right)\right)Q\left(x,dy\right)\;+f\left(x\right)\int_{G}\left(c\left(x,y\right)-b\left(x,y\right)\right)Q\left(x,dy\right).$$

In the first case of (20), we have for all x∈B that

$${\left\|M_{b}\left(x,\cdot \right)-M_{c}\left(x,\cdot \right)\right\|}_{\mathrm{tv}}\leq 2\int_{G}\left|b\left(x,y\right)-c\left(x,y\right)\right|Q\left(x,dy\right)\leq 2\int_{B}b\left(x,y\right)\xi \left(x\right)\left(\eta \left(x\right)+\eta \left(y\right)\right)Q\left(x,dy\right)\leq 2\xi \left(x\right)\left(\eta \left(x\right)+M_{b}\left(\eta \cdot 1_{B}\right)\left(x\right)\right)\leq 2\xi \left(x\right)\left(\eta \left(x\right)+M_{b}V^{\beta }\left(x\right){\left\|\eta \cdot 1_{B}\right\|}_{\infty ,V^{\beta }}\right)\leq 4T{\left\|\xi \cdot 1_{B}\right\|}_{\infty ,V^{1-\beta }}{\left\|\eta \cdot 1_{B}\right\|}_{\infty ,V^{\beta }}V\left(x\right),$$

where we used that $\sup_{x\in G}\frac{M_{b}V\left(x\right)}{V\left(x\right)}\leq T$ implies $\sup_{x\in G}\frac{M_{b}V{\left(x\right)}^{\beta }}{V{\left(x\right)}^{\beta }}\leq T^{\beta }$ by Jensen’s inequality. Moreover, for any x∈B we obtain

$${\left\|M_{b}\left(x,\cdot \right)-M_{c}\left(x,\cdot \right)\right\|}_{V}\leq \underset{\left|f\right|\leq V}{\sup}|\int_{G}f\left(y\right)\left(b\left(x,y\right)-c\left(x,y\right)\right)Q\left(x,dy\right)\;\;+f\left(x\right)\left(\int_{G}\left(c\left(x,y\right)-b\left(x,y\right)\right)Q\left(x,dy\right)\right)|\leq \int_{G}V\left(y\right)\left|b\left(x,y\right)-c\left(x,y\right)\right|Q\left(x,dy\right)+V\left(x\right)\int_{G}\left|b\left(x,y\right)-c\left(x,y\right)\right|Q\left(x,dy\right)\leq \int_{B}V\left(y\right)b\left(x,y\right)\xi \left(x\right)\left(\eta \left(x\right)+\eta \left(y\right)\right)Q\left(x,dy\right)\;+V\left(x\right)\int_{B}b\left(x,y\right)\xi \left(x\right)\left(\eta \left(x\right)+\eta \left(y\right)\right)Q\left(x,dy\right)\leq 2{\left\|\eta \cdot 1_{B}\right\|}_{\infty }{\left\|\xi \cdot 1_{B}\right\|}_{\infty }\left(M_{b}V\left(x\right)+V\left(x\right)\right),$$

which implies the assertion in that case. In the second case of (20), we have similarly for any x∈B that

$$\;{\left\|M_{b}\left(x,\cdot \right)-M_{c}\left(x,\cdot \right)\right\|}_{\mathrm{tv}}\leq 2\eta \left(x\right)M_{b}\left(\xi \cdot 1_{B}\right)+2M_{b}\left(\xi \cdot \eta \cdot 1_{B}\right)\leq 2\eta \left(x\right){\left\|\xi \cdot 1_{B}\right\|}_{\infty ,V^{1-\beta }}M_{b}\left(V^{1-\beta }\right)\left(x\right)+2{\left\|\xi \cdot \eta \cdot 1_{B}\right\|}_{\infty ,V}M_{b}V\left(x\right)\leq 4T{\left\|\eta \cdot 1_{B}\right\|}_{\infty ,V^{\beta }}{\left\|\xi \cdot 1_{B}\right\|}_{\infty ,V^{1-\beta }}V\left(x\right)$$

and

$${\left\|M_{b}\left(x,\cdot \right)-M_{c}\left(x,\cdot \right)\right\|}_{V}\leq \int_{B}V\left(y\right)b\left(x,y\right)\xi \left(y\right)\left(\eta \left(x\right)+\eta \left(y\right)\right)Q\left(x,dy\right)\;+V\left(x\right)\int_{B}b\left(x,y\right)\xi \left(y\right)\left(\eta \left(x\right)+\eta \left(y\right)\right)Q\left(x,dy\right)\leq 2{\left\|\xi \cdot 1_{B}\right\|}_{\infty }{\left\|\eta \cdot 1_{B}\right\|}_{\infty }\left(M_{b}V\left(x\right)+V\left(x\right)\right),$$

which finishes the proof. □

Before we come to further proofs of Section 4 we provide some properties of inverse moments of averages of non-negative real-valued iid random variables ${\left(S_{i}\right)}_{i\in N}$. In this setting, the pth inverse moment, for p>0, is defined by

$$j_{p,r}≔{\left(E{\left(\frac{1}{r}\overset{r}{\underset{i=1}{\sum }}S_{i}\right)}^{-p}\right)}^{1∕p}.$$

Lemma 23 *Assume that* $j_{p,r}<\infty$ *for some* r∈N *and* p>0*. Then*

(i) $j_{p,s}\leq j_{p,r}$ *for* s∈N *with* s≥r*;*
(ii) $j_{q,r}\leq j_{p,r}$ *for* 0<q<p*;*
(iii) $j_{k\cdot p,k\cdot r}\leq j_{p,r}$ *for any* k∈N*.*

Proof Properties (i), (ii) follow as in [14, Lemma 3.5]. For proving (iii) we have to show that

$$E\left[{\left(\frac{1}{k\cdot r}\overset{k\cdot r}{\underset{i=1}{\sum }}S_{i}\right)}^{-p\cdot k}\right]\leq E{\left[{\left(\frac{1}{r}\overset{r}{\underset{i=1}{\sum }}S_{i}\right)}^{-p}\right]}^{k}.$$

To this end, observe first that we can write

$$\frac{1}{k\cdot r}\overset{k\cdot r}{\underset{i=1}{\sum }}S_{i}=\frac{1}{k}\overset{k}{\underset{i=1}{\sum }}V_{i}$$

where the “batch-means” $V_{1},\ldots ,V_{k}$ are non-negative, real-valued iid random variables which have the same distribution as $\frac{1}{r}\sum_{i=1}^{r}S_{i}$. With $Z_{i}=V_{i}^{-1}$ we obtain

$$E\left[{\left(\frac{1}{\frac{1}{k\cdot r}\overset{k\cdot r}{\underset{i=1}{\sum }}S_{i}}\right)}^{p\cdot k}\right]=E\left[{\left(\frac{1}{\frac{1}{k}\overset{k}{\underset{i=1}{\sum }}\frac{1}{Z_{i}}}\right)}^{p\cdot k}\right]$$

which is a moment of the harmonic mean of $Z_{1},\ldots ,Z_{k}$. Using the inequality between geometric and harmonic means as well as the independence we find that

$$E\left[{\left(\frac{1}{\frac{1}{k}\overset{k}{\underset{i=1}{\sum }}\frac{1}{Z_{i}}}\right)}^{p\cdot k}\right]\leq E\left[\overset{k}{\underset{i=1}{\prod }}Z_{i}^{p}\right]=E{\left[Z_{1}^{p}\right]}^{k}=E{\left[{\left(\frac{1}{\frac{1}{r}\overset{r}{\underset{i=1}{\sum }}S_{i}}\right)}^{p}\right]}^{k}.\;□$$

The previous lemma shows that when inverse moments of some positive order are finite, then so are inverse moments of all higher and lower orders if the sample size is adjusted accordingly.

Proof of Lemma 14 It is easily seen that

$$a\left(x,z\right)E\left[\min\left\{1,\frac{W_{N}\left(x\right)}{W_{N}\left(z\right)}\right\}\right]\leq a_{N}\left(x,z\right)$$

for any x,z∈G. By virtue of Jensen’s inequality and $E\left[W_{N}\left(z\right)\right]=1$ we have $E\left[W_{N}{\left(z\right)}^{-1}\right]\geq 1$ as well as

$$a_{N}\left(x,z\right)\leq \min\left\{1,r\left(x,z\right)\cdot E\left[\frac{W_{N}\left(x\right)}{W_{N}\left(z\right)}\right]\right\}\leq a\left(x,z\right)$$

where we also used the independence of $W_{N}\left(x\right)$ and $W_{N}\left(z\right)$ in the last inequality. (The previous arguments are similar to those in [14, Lemma 3.3 and the proof of Lemma 3.2].) Note that $i_{2,N}\left(x\right)\leq i_{2,k}\left(x\right)$ for N≥k by Lemma 23. Hence, one can conclude that

$$\left|a\left(x,z\right)-a_{N}\left(x,z\right)\right|\leq a\left(x,z\right)\left\{\begin{array}{cc} E\left[\max\left\{0,1-\frac{W_{N}\left(x\right)}{W_{N}\left(z\right)}\right\}\right]\; & a\left(x,z\right)\geq a_{N}\left(x,z\right)\\ E\left[\frac{W_{N}\left(x\right)}{W_{N}\left(z\right)}-1\right]\; & a\left(x,z\right)<a_{N}\left(x,z\right) \end{array}\right)\;\leq a\left(x,z\right)E\left|1-\frac{W_{N}\left(x\right)}{W_{N}\left(z\right)}\right|\leq a\left(x,z\right)\;i_{2,N}\left(z\right){\left(E{\left|W_{N}\left(x\right)-W_{N}\left(z\right)\right|}^{2}\right)}^{1∕2}\;\;\leq a\left(x,z\right)i_{2,N}\left(z\right)\left[{\left(E{\left|W_{N}\left(x\right)-1\right|}^{2}\right)}^{1∕2}+{\left(E{\left|W_{N}\left(z\right)-1\right|}^{2}\right)}^{1∕2}\right]\;\leq a\left(x,z\right)\frac{i_{2,k}\left(z\right)}{\sqrt{N}}\left(s\left(x\right)+s\left(z\right)\right).\;□$$

Proof of Lemma 19 As in the previous proof or from [14, Lemma 3.3 and the proof of Lemma 3.2] an immediate consequence is

$$a\left(x,z\right)E\left[\min\left\{1,\frac{W_{N}\left(z\right)}{W_{N}\left(x\right)}\right\}\right]\leq a_{N}\left(x,z\right)\leq a\left(x,z\right)\;E\left[\frac{W_{N}\left(z\right)}{W_{N}\left(x\right)}\right].$$

Note that $i_{2,N}\leq i_{2,k}$ for N≥k, see Lemma 23. The rest of the lemma follows as in the previous proof, only the ratio $W_{N}\left(x\right)∕W_{N}\left(z\right)$ is reversed. □

Proof of Proposition 20 For random-walk-based Metropolis chains (in particular for Q as assumed in the statement) by [9, Theorem 4.1 and the first sentence after the proof of the theorem, as well as, Theorem 4.3, Theorem 4.6] we have that $M_{a}$ is $V_{t}$-uniformly ergodic with

$$V_{t}\left(x\right)\propto \pi_{u}{\left(x\right)}^{-t}\propto \exp\left(t\;\frac{\gamma_{Z,Y}}{2}{\left(z-x\right)}^{2}\right),$$

for any $t\in \left(0,1\right)$. Hence, Assumption 11 is satisfied and we need to find t∈(0,1) as well as β∈(0,1) such that ${\left\|i_{2,k}\right\|}_{\infty ,V_{t}^{1-\beta }}<\infty$ and ${\left\|s\right\|}_{\infty ,V_{t}^{\beta }}<\infty$ for some k∈N. For showing ${\left\|s\right\|}_{\infty ,V_{t}^{\beta }}<\infty$ we use (26) to see that

$$s\left(x\right)\leq \overset{˜}{C}\exp\left(\left(\frac{\gamma_{Z}}{\gamma_{Y}+2\gamma_{Z}}\right)\frac{\gamma_{Z,Y}}{2}{\left(z-x\right)}^{2}\right),$$

for some $\overset{˜}{C}<\infty$. Hence

$$\frac{s\left(x\right)}{V_{t}{\left(x\right)}^{\beta }}\leq \overset{˜}{C}\exp\left(\left(\frac{\gamma_{Z}}{\gamma_{Y}+2\gamma_{Z}}-t\beta \right)\frac{\gamma_{Z,Y}}{2}{\left(z-x\right)}^{2}\right),$$

and choosing β∈(0,1) such that

(A.4) $$t\beta =\frac{\gamma_{Z}}{\gamma_{Y}+2\gamma_{Z}}$$

leads to ${\left\|s\right\|}_{\infty ,V_{t}^{\beta }}<\infty$. In order to show ${\left\|i_{2,k}\right\|}_{\infty ,V_{t}^{1-\beta }}<\infty$, we first use Lemma 23(iii) and obtain for any x∈G and any k∈N

$$i_{2,k}\left(x\right)=E{\left[W_{k}{\left(x\right)}^{-2}\right]}^{\frac{1}{2}}\leq E{\left[W_{1}{\left(x\right)}^{-\frac{2}{k}}\right]}^{\frac{k}{2}}.$$

Then, for $k>2\gamma_{Z}∕\gamma_{Y}$ by (26) we have

$$E{\left[W_{1}{\left(x\right)}^{-\frac{2}{k}}\right]}^{\frac{k}{2}}\propto \exp\left(\left(\frac{\gamma_{Z}\left(1+\frac{2}{k}\right)}{\gamma_{Y}-\frac{2}{k}\gamma_{Z}}\right)\frac{\gamma_{Z,Y}}{2}{\left(z-x\right)}^{2}\right).$$

Therefore, there is a constant $\overset{˜}{C}<\infty$ such that

$$\frac{i_{2,k}\left(x\right)}{V_{t}{\left(x\right)}^{1-\beta }}\leq \overset{˜}{C}\exp\left(\left(\frac{\gamma_{Z}\left(1+\frac{2}{k}\right)}{\gamma_{Y}-\frac{2}{k}\gamma_{Z}}-t\left(1-\beta \right)\right)\frac{\gamma_{Z,Y}}{2}{\left(z-x\right)}^{2}\right).$$

We have ${\left\|i_{2,k}\right\|}_{\infty ,V_{t}^{1-\beta }}<\infty$ if $\frac{\gamma_{Z}\left(1+\frac{2}{k}\right)}{\gamma_{Y}-\frac{2}{k}\gamma_{Z}}\leq t\left(1-\beta \right)$. The latter condition holds whenever

$$k\geq \frac{2\gamma_{Z}\left(1+t\left(1-\beta \right)\right)}{\gamma_{Y}t\left(1-\beta \right)-\gamma_{Z}},$$

provided that $t\left(1-\beta \right)>\gamma_{Z}∕\gamma_{Y}$. This implies, by (A.4), that t should be chosen such that

(A.5) $$t>\frac{\gamma_{Z}}{\gamma_{Y}}+\frac{\gamma_{Z}}{\gamma_{Y}+2\gamma_{Z}}.$$

Choosing t such that it satisfies (A.5) is feasible whenever the right-hand side of (A.5) is smaller than 1. This is the case if $\gamma_{Y}>\sqrt{2}\gamma_{Z}$. □
